# ﻿A revision of the genus *Ecpyrrhorrhoe* Hübner, 1825 from China based on morphology and molecular data, with descriptions of five new species (Lepidoptera, Crambidae, Pyraustinae)

**DOI:** 10.3897/zookeys.1090.78442

**Published:** 2022-03-23

**Authors:** Lanbin Xiang, Kai Chen, Xiaohua Chen, Yongjiang Duan, Dandan Zhang

**Affiliations:** 1 School of Life Sciences, Sun Yat-sen University, Guangzhou, Guangdong 510275, China Sun Yat-sen University Guangzhou China; 2 School of Ecology, Sun Yat-sen University, Guangzhou, Guangdong 510275, China Sun Yat-sen University Guangzhou China

**Keywords:** Molecular phylogeny, morphology, new combinations, new synonyms, *Yezobotys*

## Abstract

The genus *Ecpyrrhorrhoe* Hübner, 1825 is revised. Most type materials were examined, and a preliminary phylogeny is presented based on a combined dataset of COI, 16S rRNA, 28S rRNA and EF-1α gene regions. The tree topology and morphological characters suggest that *Paliga* Moore, 1886 is a new synonym of *Ecpyrrhorrhoe*, and *Yezobotys* Munroe & Mutuura, 1969 is restored as a valid genus. According to the morphological evidence and tree topology, 17 species are recorded, including five new species described from China: *E.allochroa* Zhang & Xiang, **sp. nov.**, *E.rosisquama* Xiang & Zhang, **sp. nov.**, *E.exigistria* Zhang & Xiang, **sp. nov.**, *E.brevis* Zhang & Xiang, **sp. nov.** and *E.longispinalis* Zhang & Xiang, **sp. nov.** Seven new combinations are created, *E.damastesalis* (Walker, 1859), **comb. nov.**, *E.minnehaha* (Pryer, 1877), **comb. nov.**, *E.obliquata* (Moore, 1888), **comb. nov.**, *E.rufipicta* (Butler, 1880), **comb. nov.**, *E.fimbriata* (Moore, 1886), **comb. nov.**, *E.machoeralis* (Walker, 1859), **comb. nov.**, and *E.rubellalis* (Snellen, 1890), **comb. nov.**, as well as eight new synonyms, namely *Leucocraspedaauratalis* Warren, 1895, **syn. nov.**, *Pioneaschenklingi* Strand, 1918, **syn. nov.**, *Paligarubicundalis* Warren, 1896, **syn. nov.**, *E.angustivalvaris* Gao, Zhang & Wang, 2013, **syn. nov.**, *Pyraustapygmaealis* South, 1901, **syn. nov.**, *E.multispinalis* Gao, Zhang &Wang, 2013, **syn. nov.**, *E.aduncis* Gao, Zhang & Wang, 2013, **syn. nov.**, and *E.ruidispinalis* Zhang, Li & Wang, 2004, **syn. nov.** All adults and their genital structures are illustrated and an identification key based on adult external morphology and genitalia is provided.

## ﻿Introduction

The genus *Ecpyrrhorrhoe* Hübner, 1825, type species *Pyralisrubiginalis* Hübner, 1796, was regarded as monotypic from its original description until Maes (1994) synonymized *Harpadispar* Agenjo, 1952 with this genus. Later, records by Zhang et al. (2004), Zhang and Li (2008), Solis et al. (2010), Gao et al. (2013), Maes (2014), and Nuss et al. (2003–2022) show that this genus is more diverse and widely distributed than previously thought. Six new species are described, and four species are transferred to *Ecpyrrhorrhoe* in these studies. Additionally, Solis et al. (2010) treated *Yezobotys* Munroe & Mutuura, 1969 as a synonym of *Ecpyrrhorrhoe*. Thus, the species number of *Ecpyrrhorrhoe* was increased to 12.

There are striking apomorphic characters available to diagnose species of *Ecpyrrhorrhoe*. These are a narrowly lanceolate uncus, long dorsolateral arms of the juxta, the presence of spines on the anellus, a slender longitudinal sclerite located in the posterior part of the ductus bursae, and a second (posterior) signum with spines in the female genitalia. *Paliga* Moore, 1886 shares some of these characters with species of *Ecpyrrhorrhoe*, but *Yezobotys* does not share these characters. Therefore, the relationship of *Yezobotys* and *Paliga* with *Ecpyrrhorrhoe* needs to be resolved.

Upon examination of pyraustine collections from China, and type specimens and other material from the Natural History Museum, London, United Kingdom, and the Senckenberg Entomological Institute, Brandenburg, Germany, some known species, and undescribed species were found to agree with the circumscription of *Ecpyrrhorrhoe* based on genitalia characters. In order to evaluate the generic placements of these species and the taxonomic composition of *Ecpyrrhorrhoe*, the phylogenetic relationships of *Ecpyrrhorrhoe* were studied with molecular data.

## ﻿Materials and methods

### ﻿Molecular phylogenetic analysis

In total 24 species were included in the molecular phylogenetic analysis (Table 1), including the type species of *Yezobotys* and *Paliga*, five new species and six putative new combinations. *Euclastastoetzneri* (Caradja, 1927) was chosen as the outgroup because it has been inferred as sister-group of the Pyraustini and Portentomorphini in Pyraustinae (Mally et al. 2019). Two species of *Pyrausta* Schrank, 1802, two species of *Pagyda* Walker, 1859, and two species of *Anamalaia* Munroe & Mutuura, 1969 were included as related taxa because of the similar external and genital characters, and a previous taxonomic treatment of *Ecpyrrhorrhoe* as a subgenus of *Pyrausta* (Hannemann 1964).

**Table 1.** Species sampled for the molecular phylogenetic analyses; all species sequenced in this study except *Euclastastoetzneri*, which was sequenced by Zhang et al. (2020).

Genus	Species	Voucher	Locality	GenBank accession number			
				COI	16S	EF-1α	28S
*Euclasta*	*stoetzneri*	SYSULEP0334	Shaanxi	MT738696	MT734412	MT724335	MT734404
*Yezobotys*	*dissimilis*	SYSULEP0029	Hubei	OM674485	OM672201	OM650166	OM672234
*Yezobotys*	*dissimilis*	SYSULEP0089	Hubei	OM674486	OM672202	N/A	N/A
*Anamalaia*	*lutusalis*	SYSULEP0088	Yunnan	OM674487	OM672205	OM650169	OM672237
*Anamalaia*	*fasciata*	SYSULEP0233	Yunnan	OM674488	OM672206	OM650170	OM672238
*Pagyda*	*salvalis*	SYSULEP0086	Yunnan	OM674489	OM672203	OM650167	OM672235
*Pagyda*	*recticlavata*	SYSULEP0091	Jiangxi	OM674490	OM672204	OM650168	OM672236
*Pyrausta*	*panopealis*	SYSULEP0072	Jiangxi	OM674491	OM672199	OM650164	OM672232
*Pyrausta*	*despicata*	SYSULEP0348	Xinjiang	OM674492	OM672200	OM650165	OM672233
*Ecpyrrhorrhoe*	*biaculeiformis*	SYSULEP0015	Hunan	OM674493	OM672174	OM650146	OM672213
*Ecpyrrhorrhoe*	*digitaliformis*	SYSULEP0016	Hubei	OM674494	OM672175	OM650147	OM672214
*Ecpyrrhorrhoe*	*celatalis*	SYSULEP0017	Hainan	OM674495	OM672176	OM650148	OM672215
*Ecpyrrhorrhoe*	*rubiginalis*	SYSULEP0019	Shanxi	OM674496	OM672177	N/A	N/A
*Ecpyrrhorrhoe*	*rosisquama*	SYSULEP0020	Yunnan	OM674497	OM672178	OM650149	OM672216
*Ecpyrrhorrhoe*	*rubellalis*	SYSULEP0023	Hainan	OM674498	OM672179	N/A	OM672217
*Ecpyrrhorrhoe*	*biaculeiformis*	SYSULEP0024	Hubei	OM674499	OM672180	N/A	N/A
*Ecpyrrhorrhoe*	*obliquata*	SYSULEP0034	Hainan	OM674500	OM672181	OM650150	OM672218
*Ecpyrrhorrhoe*	*damastesalis*	SYSULEP0035	Yunnan	OM674501	OM672182	N/A	N/A
*Ecpyrrhorrhoe*	*brevis*	SYSULEP0036	Guangdong	OM674502	OM672183	OM650151	OM672219
*Ecpyrrhorrhoe*	*puralis*	SYSULEP0037	Hunan	OM674503	OM672184	OM650152	OM672220
*Ecpyrrhorrhoe*	*rubiginalis*	SYSULEP0048	Shanxi	OM674504	OM672185	OM650153	N/A
*Ecpyrrhorrhoe*	*longispinalis*	SYSULEP0058	Hunan	OM674505	OM672186	OM650154	OM672221
*Ecpyrrhorrhoe*	*minnehaha*	SYSULEP0059	Jiangxi	OM674506	OM672187	OM650155	OM672222
*Ecpyrrhorrhoe*	*allochroa*	SYSULEP0060	Hainan	OM674507	OM672188	N/A	OM672223
*Ecpyrrhorrhoe*	*minnehaha*	SYSULEP0061	Jiangxi	OM674508	OM672189	N/A	N/A
*Ecpyrrhorrhoe*	*rufipicta*	SYSULEP0062	Hainan	OM674509	OM672190	N/A	OM672224
*Ecpyrrhorrhoe*	*exigistria*	SYSULEP0063	Yunnan	OM674510	OM672191	OM650156	OM672225
*Ecpyrrhorrhoe*	*exigistria*	SYSULEP0100	Jiangxi	OM674511	OM672192	OM650157	N/A
*Ecpyrrhorrhoe*	*fimbriata*	SYSULEP0111	Yunnan	OM674512	N/A	N/A	N/A
*Ecpyrrhorrhoe*	*rufipicta*	SYSULEP0107	Hainan	OM674513	OM672193	OM650158	OM672226
*Ecpyrrhorrhoe*	*rubiginalis*	SYSULEP0109	Jiangxi	OM674514	OM672194	OM650159	OM672227
*Ecpyrrhorrhoe*	*rubellalis*	SYSULEP0110	Guangxi	OM674515	N/A	OM650160	N/A
*Ecpyrrhorrhoe*	*damastesalis*	SYSULEP0163	Yunnan	OM674516	OM672195	OM650161	OM672228
*Ecpyrrhorrhoe*	*exigistria*	SYSULEP0211	Guangxi	OM674517	OM672196	OM650162	OM672229
*Ecpyrrhorrhoe*	*minnehaha*	SYSULEP0217	Guangdong	OM674518	OM672197	OM650163	OM672230
*Ecpyrrhorrhoe*	*obliquata*	SYSULEP0297	Guangdong	OM674519	OM672198	N/A	OM672231

Total DNA was extracted from two legs and sometimes additionally from the abdomen of the dry specimens using the TIANGEN DNA extraction kit following the manufacturer’s instructions. The nucleotide sequences of two mitochondrial genes, cytochrome c oxidase subunit I (COI) and 16S ribosomal RNA (16S rRNA), and two nuclear genes, 28S ribosomal RNA (28S rRNA) and Elongation factor-1 alpha (EF-1α) were selected for study. Primers used in this study and all PCRs performed follow Zhang et al. (2020). PCR products were confirmed with 1.5% agarose gel electrophoresis in TAE buffer, then were direct-sequenced at Majorbio Bio-pharm Technology Co., Ltd (Guangzhou), utilizing the same primers used for PCR amplification.

The sequences were aligned using Clustal W (Thompson et al. 1994) in MEGA 6 (Tamura et al. 2013) with default settings. The aligned matrix was corrected by eye. Gaps were treated as missing data. Phylogenetic analyses were inferred using Bayesian inference (BI) method in MrBayes 3.2.6 (Ronquist et al. 2012) and maximum likelihood (ML) in RAxML 8.2.10 (Stamatakis 2014). BI analysis was run with independent parameters all under the GTR + G + I model for four gene partitions, as suggested by jModelTest 0.1.1 (Posada 2008). Two independent runs, each with four Markov Chain Monte Carlo (MCMC) simulations, were performed for 20 million generations sampled every 1000^th^ generation. The first 25% trees were discarded as burn-in, and posterior probabilities (PP) were determined from remaining trees. The ML analysis was executed under the GTR + G + I model for all gene partitions and with 1000 iterations for the bootstrap test. The bootstrap value (BS) ≥ 90 is considered absolute support, 75 ≤ BS < 90 is considered strong support, and 50 ≤ BS < 75 is considered weak support. PP ≥ 95 is considered strong support and 80 ≤ PP < 95 is considered weak support. The pairwise Kimura 2-Parameter (K2P) distances between species were calculated from the COI gene using MEGA 6 (Tamura et al. 2013).

### ﻿Morphological analysis

The specimens studied, including the types of the newly described species, are deposited in the Museum of Biology, Sun Yat-sen University, Guangzhou, China (**SYSBM**), except for those held at the following institutions: Insect Collection of the College of Life Sciences, Nankai University, China (**NKU**), Natural History Museum, London, United Kingdom (**NHMUK**) and Senckenberg Deutsches Entomologisches Institut, Brandenburg, Germany (**SDEI**). Slides of dissected genitalia were prepared according to the protocols of Robinson (1976) and Li and Zheng (1996). Terminology of genitalia follows Maes (1995), except for “phallus” and “colliculum” for which we follow Kristensen (2003). Images of the specimens were taken using a Canon EOS 80D camera provided with a Canon 100 mm macro lens. The genitalia photographs were taken using a Zeiss Axio Scope.A1 in combination with a Zeiss AxioCam camera and the Axio Vision SE64 program on a Windows PC. Source images were then aligned and stacked with Helicon Focus to obtain a composite image. All the pictures were edited using Adobe Photoshop SC5.

## ﻿Results

### ﻿Phylogenetic relationships

The concatenated dataset of four genes consisted of 2503 nucleotide positions (657 for COI, 471 for 16S rRNA, 610 for 28S rRNA, and 765 for EF-1α). Pairwise distances of the barcoding region (COI) are given in Suppl. material 1. The genetic distances between *Ecpyrrhorrhoe* and other genera range from 8.5% (*Yezobotys*) to 15.0% (*Euclasta*). Interspecific genetic distances within *Ecpyrrhorrhoe* range from 6.6% (*E.celetalis* to *E.brevis*) to 14.3% (*E.damastesalis* to *E.rosisquama*), while intraspecific genetic distances in *Ecpyrrhorrhoe* range from 0% (*E.biaculeiformis*, *E.rufipicta*, *E.minnehaha*, and *Yezobotysdissimilis*) to 1.2% (*E.exigistria*).

**Figure 1.**
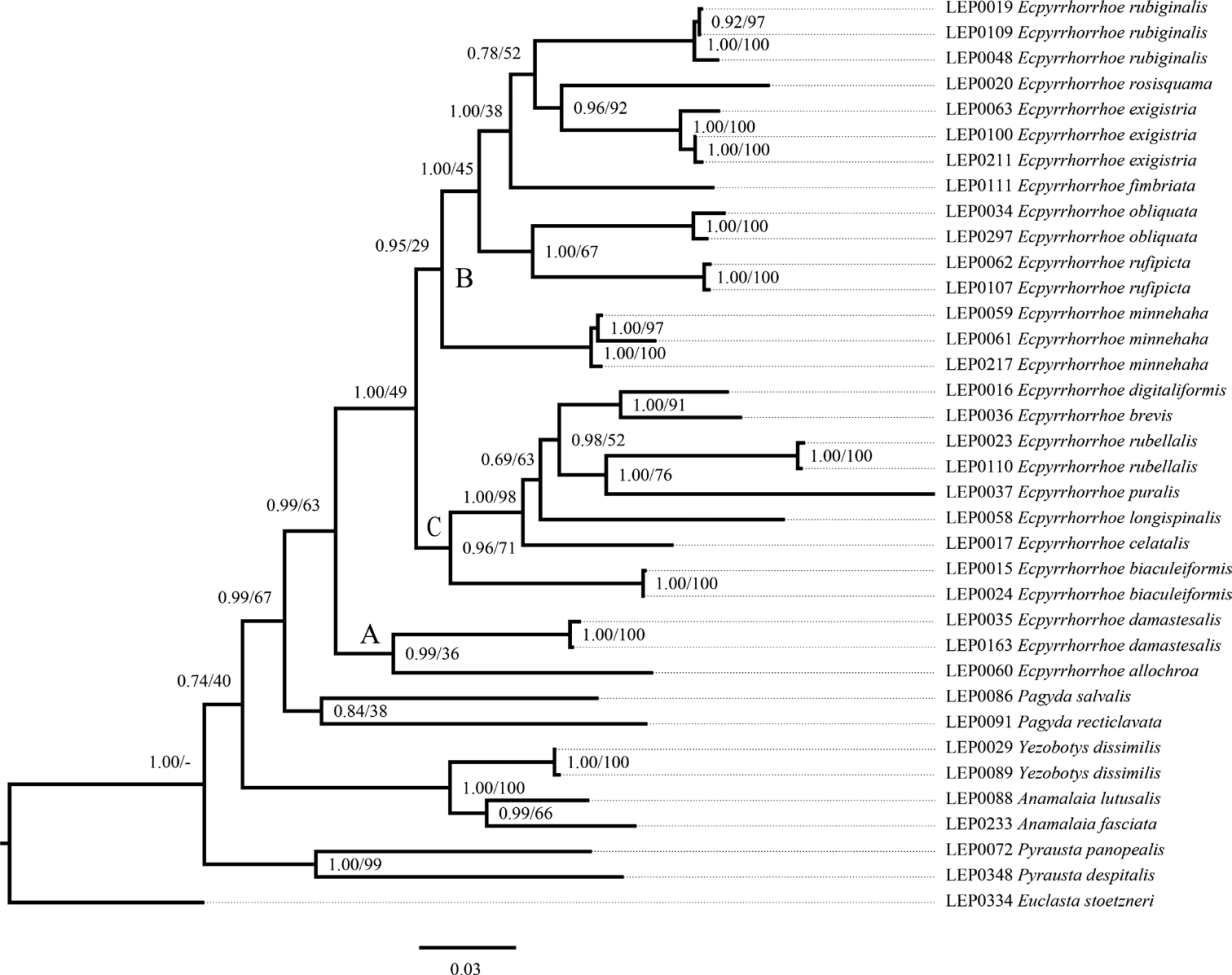
Phylogenetic hypothesis inferred from the BI analysis. Numbers on branches indicate Bayesian posterior probabilities and ML bootstrap values, respectively.

Both BI and ML analyses of the concatenated dataset inferred fully congruent relationships with only subtle differences in posterior probability and bootstrap values (Fig. 1). The tree topology indicates that *Yezobotys* Munroe & Mutuura, 1969 should be restored as a valid genus because the type species, *Y.dissimilis* (Yamanaka, 1958), appears as sister to *Anamalaia* (PP = 1, BS = 100). The monophyly of *Ecpyrrhorrhoe* is supported (PP = 0.99, BS = 63). The genus *Pagyda* Walker, 1859 is the sister group of *Ecpyrrhorrhoe* (PP = 0.99, BS = 67). Among *Ecpyrrhorrhoe* species included in the analysis, the majority of the basal nodes are strongly supported in BI but relatively poorly supported in ML.

According to the tree topology and morphological characters, the genus *Ecpyrrhorrhoe* can be divided into three species groups (The A clade, B clade, and C clade). The A clade is the sister group to B clade + C clade (PP = 0.99, BS = 63). The A clade is composed of *E.allochroa* and *E.damastesalis*. The B clade consists of *E.minnehaha*, *E.rufipicta*, *E.obliquata*, *E.fimbriata*, *E.rubiginalis*, *E.exigistria*, and *E.rosisquama*, in which *E.minnehaha* is the basal taxon, and *E.rufipicta* + *E.obliquata* is the sister group to *E.fimbriata* + (*E.rubiginalis* + (*E.exigistria* + *E.rosisquama*)) (PP = 1.00, BS = 45). The C clade is composed of seven species, namely *E.biaculeiformis*, *E.celatalis*, *E.longispinalis*, *E.puralis*, *E.rubellalis*, *E.brevis* and *E.digitaliformis*, in which *E.biaculeiformis* is the basal taxon, and *E.celatalis* is the sister group with the clade *E.longispinalis* + ((*E.puralis* + *E.rubellalis*) + (*E.brevis* + *E.digitaliformis*)) (PP = 1.00, BS = 98). The sister groups *E.exigistria* and *E.rosisquama* (PP = 0.96, BS = 92), *E.obliquata* and *E.rufipicta* (PP = 1.00, BS = 67), *E.digitaliformis* and *E.brevis* (PP = 1.00, BS = 91), and *E.rubellalis* and *E.puralis* (PP = 1.00, BS = 76) are supported in both BI and ML.

The results of the molecular phylogenetic analyses support the placement of five undescribed species (named as *E.allochroa* sp. nov., *E.rosisquama* sp. nov., *E.exigistria* sp. nov., *E.brevis* sp. nov., and *E.longispinalis* sp. nov.) in *Ecpyrrhorrhoe*, the transfer of *E.rubellalis* (Snellen, 1890) , comb. nov. from *Pyrausta* Schrank, 1802 to *Ecpyrrhorrhoe*, the transfer of *E.obliquata* (Moore, 1888), comb. nov. and *E.fimbriata* (Moore, 1886), comb. nov. from *Anania* Hübner, 1823 to *Ecpyrrhorrhoe*, and the transfer of *E.damastesalis* (Walker, 1859), comb. nov., *E.rufipicta* (Butler, 1880), comb. nov., and *E.minnehaha* (Pryer, 1877), comb. nov. from *Paliga* Moore, 1886 to *Ecpyrrhorrhoe*. The taxonomic details are given below.

## ﻿Taxonomy

### ﻿ Ecpyrrhorrhoe

Taxon classificationAnimaliaLepidopteraCrambidae

Hübner, 1825

4C0DD02B-7211-51D8-B69A-760F0C9E76E0

Ecpyrrhorrhoe Hübner, 1825. Type species: Pyralisrubiginalis Hübner, 1796, by subsequent designation by Hannemann, 1964.
Ecpyrrhorrhoea Hübner, 1825. Misspelling.
Ecpyrrhorrhoa Agassiz, 1846. Misspelling.
Paliga Moore, 1886. Type species: Scopuladamastesalis Walker, 1859, by monotypy. Syn. nov.
Eutectona Wang & Sung, 1980. Type species: Scopulamachoeralis Walker, 1859, by original designation.
Harpadispar Agenjo, 1952. Type species: Botysdiffusalis Guenée, 1854, by original designation.
Pyraustegia Marion, 1963. Type species: Botysdiffusalis Guenée, 1854, by original designation.

#### Diagnosis.

The wings of species of *Ecpyrrhorrhoe* are usually yellow, sometimes decorated with pink or brown scales. In appearance, they are similar to some species of *Pyrausta* Schrank, 1802 and *Pseudopagyda* Slamaka, 2013, but can be distinguished by the usually obvious dark brown subterminal band on the underside of wings. They are characterized by the lanceolate, densely setose uncus; the mostly thumb-shaped sella extending to the ventral margin of the valva; the long arms of the bifid juxta, usually; usually the presence of several spines or sclerites on the anellus in the male genitalia. In the female genitalia, the strongly sclerotized antrum, the slender sclerite located in the posterior part of the ductus bursae, and the second (posterior) signum bearing spines are characteristic.

#### Description.

**Figure 2.**
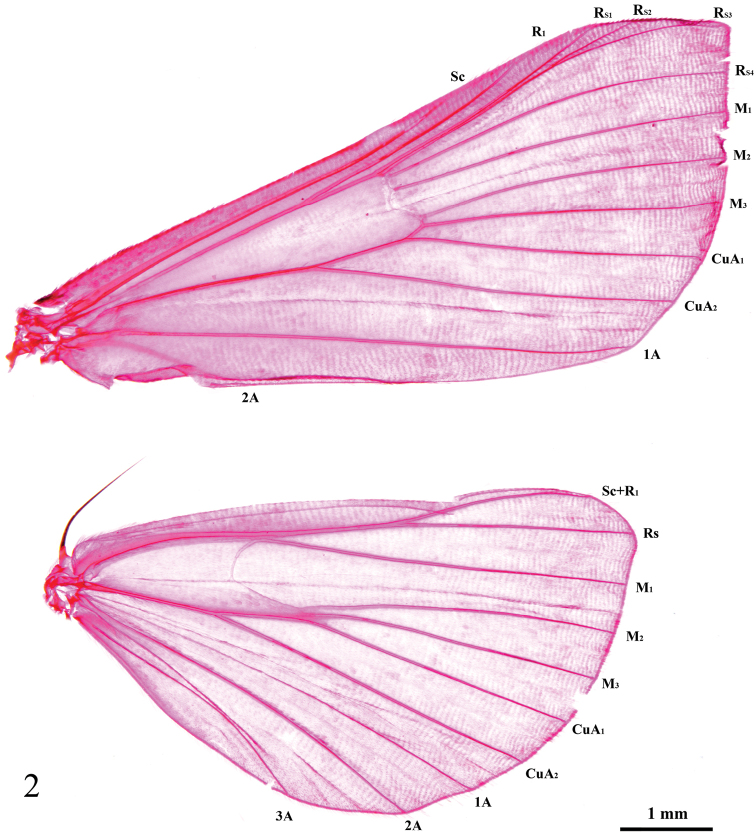
Wing venation of *Ecpyrrhorrhoerubiginalis*.

Frons oblique, smoothly scaled. Vertex with moderately raised scales projecting between antennae. Labial palpus porrect, second segment pointing obliquely upward, third segment pointing slightly downward; exceeding frons by approximately as much as length of head. Maxillary palpus small. Forewing termen gently arched. Hindwing frenulum single in male, with two acanthae in female. Wings usually yellow or yellowish brown, sometimes pink or covered with brown scales; forewing with antemedial and postmedial lines, orbicular and reniform stigma; underside of wings usually with obvious blackish brown subterminal band. Wing venation as in Fig. 2.

#### Male genitalia.

Uncus with lanceolate head and a nearly triangular, broad, naked base; densely covered with short simple, thick, setae, and every cluster of setae is made up of two independent setae. Transtilla inferior thin and stick-shaped. Valva elongated tongue-shaped; costa straight to concave; sella thumb-shaped or semicircular, extending to ventral margin of valva, usually with dense setae or spines; sacculus broad, usually with a wide dorsal protrusion. Dorsolateral arms of juxta usually long and tapering; anellus with several spines. Saccus nearly triangular. Phallus tubular.

#### Female genitalia.

Ovipositor lobes densely setose. Anterior apophysis usually ~ 1.5–2.5× the length of posterior apophysis. Antrum usually cup-shaped, sometimes densely spinulose; the ductus seminalis inserting to anterior end of colliculum, sometimes wide and sclerotized at base; ductus bursae long and slender, posterior part with a slender sclerite; corpus bursae globular, appendix bursae arising from anterior part of corpus bursae; signum (anterior-most signum) rhombic, second signum (posterior signum) strongly sclerotized and bearing spines.

#### Distribution.

Asia, Europe, South Africa, Australia, North America.

### ﻿Key to studied species of *Ecpyrrhorrhoe*

**Table:** 

1	Hindwing without postmedial line	**2**
–	Hindwing with postmedial line	**3**
2	Forewing without rosy scales (Fig. 3); excurved sella with longer setae ventrally; anellus with a long and curved, densely spinulose sclerite (Fig. 22)	***E.allochroa***
–	Forewing usually bearing rosy markings and scales on veins (Figs 4, 5); straight sella bearing short spines ventrally; anellus with three groups of spines (Fig. 23)	***E.damastesalis***
3	Forewing with covering of rosy-red scales or some specimens with rosy-red forewing	**4**
–	Forewing pale yellow, yellow, or yellowish brown	**5**
4	Forewing covering rosy-red scales; straight sella extending ventrad, bearing short spines on distal margin (Fig. 13); distal end of phallus not swirly; anellus with two groups of short and pointed spines (Fig. 29); inner wall of antrum without minute spines (Fig. 44)	***E.rosisquama***
–	Some specimens with rosy-red forewing (Figs 6, 7); excurved sella with dense covering of thick setae; distal end of phallus somewhat swirly; anellus with a cluster of short spines (Fig. 24); inner wall of antrum densely covered with minute spines (Fig. 39)	***E.minnehaha***
5	Forewing with an oblique and dark brown streak	**6**
–	Forewing without streak	**7**
6	Sella semicircular, bearing many short spines ventrally; weakly sclerotized arms of juxta without teeth (Fig. 25); antrum cup-shaped (Fig. 40)	***E.obliquata***
–	Sella short, thumb-shaped, with dense setae ventrally; strongly sclerotized arms of juxta with teeth (Fig. 30); antrum mostly tubular (Fig. 45)	***E.exigistria***
7	Sella spine-shaped or hook-shaped	**8**
–	Sella thumb-shaped, finger-shaped, or nearly triangular	**9**
8	Wings yellowish brown (Fig. 18); costa of valva somewhat straight; sella spine-shaped; phallus apically with a densely dentated, triangular cornutus; anellus with a thick spine bearing a broad and long, spinulose base (Fig. 34); antrum wrinkled medially (Fig. 49)	***E.rubellalis***
–	Wings yellow (Fig. 19); costa of valva concave; sella hook-shaped; phallus apically with a long and strong spine; anellus bearing a small and sclerotized ball, with two small spines on opposite sides (Fig. 35); antrum decorated with many small spines forming a circle (Fig. 50)	***E.longispinalis***
9	Forewing length relatively small; subterminal band of wings distinct and dark brown or brown	**10**
–	Forewing length relatively large; subterminal band of wings indistinct and yellowish brown 13
10	Fringe alternating with pale and dark brown from the base to the end (Fig. 10)	***E.fimbriata***
–	Fringe concolorous	**11**
11	Inner wall of antrum densely covered with minute spines (Fig. 41)	***E.rufipicta***
–	Inner wall of antrum without minute spines	**12**
12	Antrum with anterior 1/3 narrower than posterior 2/3 (Fig. 42)	***E.rubiginalis***
–	Antrum with anterior half narrower than posterior half (Fig. 43)	***E.machoeralis***
13	Sella almost without setae, bearing four spines; anellus with two spines between (Fig. 37)	***E.biaculeiformis***
–	Sella setose, without spine; anellus with a long, thick and large spine or a series of spines	**14**
14	Costa of valva curved	**15**
–	Costa of valva nearly straight	**16**
15	Arms of juxta bearing a small sclerotized tooth; anellus with a series of minute spines (Fig. 31)	***E.digitaliformis***
–	Arms of juxta without tooth; anellus with a series of long spines standing on a long base (Fig. 32)	***E.brevis***
16	Arms of juxta with a big tooth-like process; anellus with a long, thick and large spine (Fig. 36)	***E.celatalis***
–	Arms of juxta without process; anellus with spines appearing comb-shaped (Fig. 33)	***E.puralis***

### ﻿ Ecpyrrhorrhoe allochroa

Taxon classificationAnimaliaLepidopteraCrambidae

Zhang & Xiang sp. nov.

40AC6FC0-832F-5237-923E-D29DDF7F6CF7

http://zoobank.org/E9D003C7-8434-40EB-9961-03670363CD3D

Figs 3 , 22

#### Diagnosis.

**Figures 3–12.**
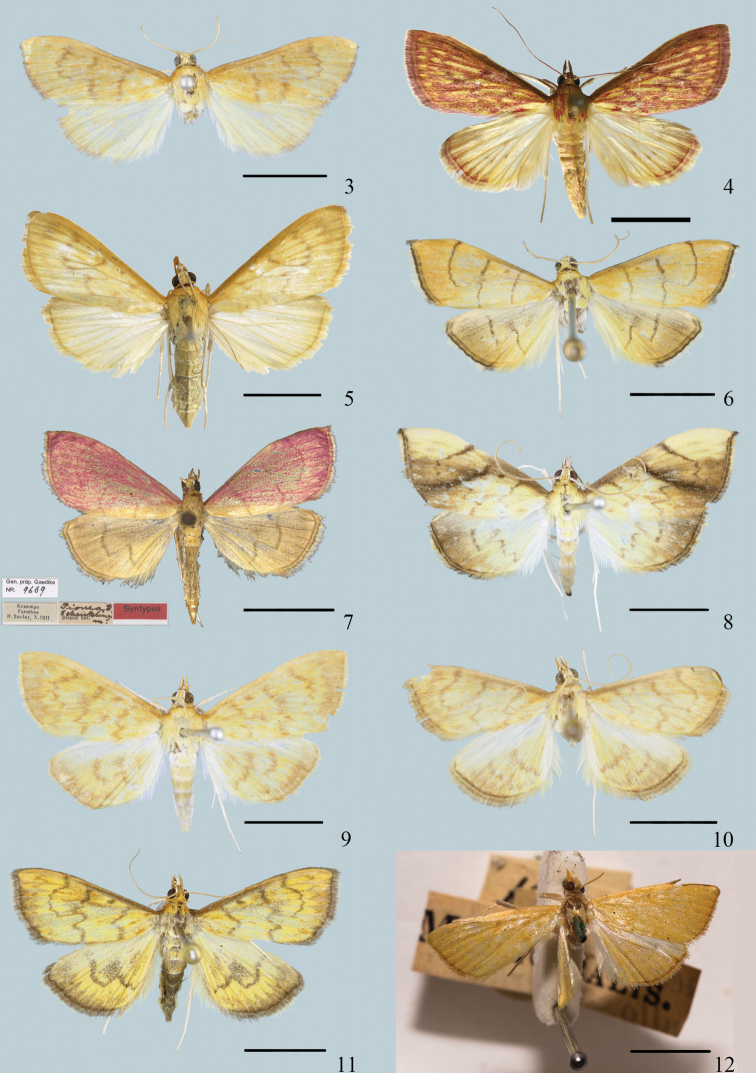
Adults of *Ecpyrrhorrhoe* spp. **3***E.allochroa*, sp. nov., holotype, male (Yunnan) **4***E.damastesalis*, male (Guangdong) **5***E.damastesalis*, male (Yunnan) **6***E.minnehaha*, male (Guangdong) **7***E.minnehaha*, male (Taiwan) **8***E.obliquata*, male (Hainan) **9***E.rufipicta*, female (Hainan) **10***E.fimbriata*, male (Guangxi) **11***E.rubiginalis*, female (Xinjiang) **12***E.machoeralis*, type, female (Sri Lanka). Scale bars: 5.0 mm.

In appearance *Ecpyrrhorrhoeallochroa* resembles *E.damastesalis* in the narrow forewing and yellowish hindwing without markings. However, it can be differentiated from *E.damastesalis* by its smaller size (forewing length: 8.0–10.0 mm), forewing scattered with yellowish brown scales and bearing brown markings (Fig. 3), in the male genitalia (Fig. 22) by the slender valva, the excurved sella with longer setae ventrally, the long and slender phallus, and long and curved, densely spinulose sclerite on anellus.

#### Description

(Fig. 3). **Head.** Frons yellow, sometimes white medially, with white lateral bands. Vertex pale yellow. Labial palpus dark yellow or yellowish brown, contrastingly white at base ventrally. Maxillary palpus yellowish brown, pale terminally. **Thorax.** Dorsal side dark yellow or yellowish brown, ventral side white. Legs white to pale yellow. **Wings.** Forewing length: 8.0–10.0 mm. Forewing pale yellow, densely scattered with yellowish brown scales, lines and stigmata brown; antemedial line from ~ 1/4 of costa to 1/2 of posterior margin; orbicular stigma oblate, sometimes weak; reniform stigma comma-shaped, slightly concave or bending inwards at middle; postmedial line from 3/4 of costa, dentated beyond basal half of CuA_1_, bending inward to 1/3 of CuA_2_, then dentated to 2/3 of posterior margin; fringe dark brown. Hindwing yellowish white, with area pale yellow; fringe pale brown and whitish at tornus. **Abdomen.** Pale dark yellow or yellowish brown dorsally, white ventrally.

#### Male genitalia

**(Fig. 22).** Uncus with basal 2/5 nearly triangular and naked. Valva slightly curved, of even width, apex obtusely rounded; sella thumb-shaped and excurved, setose, bearing several spines on ventral margin; sacculus with dorsal 3/5 inflated into a nearly triangular protrusion. Juxta shield-shaped, with base wide, distal 1/4 bifid into thick and short arms with membranous extension; anellus with a long and curved, densely spinulose sclerite (attached to distal end of phallus in Fig. 22). Saccus rounded triangular. Phallus long and slender, distal part slightly curved upward and sclerotized, with a cluster of interlaced spicules on vesica; distal end with a bent, spine-like cornutus.

#### Female genitalia.

Unknown.

#### Material examined.

**Type material. *Holotype*** ♂, **China: Yunnan**: Mengla, Xishuangbanna, 28.X.2010, Hu Bingbing, Zhang Jin, Cai Yanpeng leg., genitalia slide No. CXH12133 (SYSBM). ***Paratypes*: CHINA: Guizhou**: 1♂, Fade Bridge, Shunchang, 29.IV–3.V.2019, Liu Qingming leg., genitalia slide No. SYSU1511; **Hainan**: 1♂, Hongxin Village, Yuanmen, Baisha, 19.07°N, 109.52°E, alt. 460 m, 30.VI.2014, Cong Peixin, Liu Linjie, Hu Sha leg., genitalia slide No. ZDD12045, molecular voucher No. LEP0060 (NKU).

#### Distribution.

China (Guizhou, Hainan, Yunnan).

#### Etymology.

The specific name is derived from the Latin *allochrous* (= heterochromatic), referring to the color difference between the forewing and hindwing.

### ﻿ Ecpyrrhorrhoe damastesalis

Taxon classificationAnimaliaLepidopteraCrambidae

(Walker, 1859) comb. nov.

07694BFB-CC13-50F1-B32B-0BDCBF945D9D

Figs 4 , 5 , 23 , 38

Scopula damastesalis Walker, 1859: 1013.

#### Diagnosis.

In the male genitalia (Fig. 23), *E.damastesalis* can be characterized by the relatively thick and tapered uncus, the slender and straight sella bearing more strongly sclerotized short spines almost vertically placed on ventral and distal margins, and the three groups of spines present on anellus. The female genitalia (Fig. 38) are unique, readily separable by the triangular antrum, the wrinkled corpus bursae, the significantly large and generally subtriangular rhomboid signum with anterior and posterior parts asymmetrical and bearing a slightly curved carina, as well as the longer spines on markedly large second (posterior) signum.

#### Redescription

(Figs 4, 5). **Head.** Frons yellow, or yellowish brown scattered with rosy scales, with white lateral bands. Vertex pale yellow, usually scattered with rosy scales. Labial palpus yellowish brown or brown, usually scattered with rosy scales, contrastingly white at base ventrally. Maxillary palpus yellowish brown or brown, usually scattered with rosy scales, pale yellow terminally. **Thorax.** Dorsal side yellow, and ventral side white; tegula yellow or mixed with rosy scales sometimes. **Wings.** Forewing length: 10.0–14.0 mm. Forewing narrow and elongated; pale yellow, usually covered with rosy scales on veins, markings yellowish brown or rosy; antemedial line obliquely from 1/4 of costa to beyond posterior margin of cell, then deeply dentated to basal 1/3 of dorsum; orbicular stigma oval and distinct; reniform stigma comma-shaped, short and thick, sometimes concave; postmedial line bent inwards from 3/4 of costa, then arched and crenulated to basal half of CuA_1_, finally sharply dentated to 2/3 of posterior margin; subterminal line crenulated, sometimes faint; vein ends sometimes bearing triangular spots; fringe pale yellow, mostly mixed with rosy scales sometimes. Hindwing yellowish white, termen yellowish brown or rosy; fringe pale yellow, sometimes with some pink toward apex. **Abdomen.** Pale yellow dorsally, white ventrally.

#### Male genitalia

**(Fig. 23).** Uncus relatively thick and tapered, with basal 1/3 nearly triangular and naked. Valva slightly broad, with costa nearly straight or concave and ventral margin curved, basal part weakly narrowed, remainder of even width, apex obtusely rounded; sella thumb-shaped and straight, extending inward, bearing short spines vertically settled on ventral and distal margins; sacculus with dorsal 3/5 inflated into a nearly triangular protrusion. Juxta shield-shaped, medially weakly sclerotized, bifid; anellus with three groups of spines (attached to distal juxta in Fig. 23). Saccus narrowly triangular. Phallus long and slender, basal part slightly curved, with a bunch of interlaced spicules on vesica.

#### Female genitalia

**(Fig. 38).** Anterior apophysis ~ 2× length of posterior apophysis. Antrum subtriangular, strongly sclerotized; colliculum short; ductus bursae ~ 1.5–2× as long as diameter of corpus bursae, posterior sclerite absent. Corpus bursae globular, strongly wrinkled; rhomboid signum large, nearly triangular, with anterior and posterior parts asymmetrical, bearing slightly curved carina; second (posterior) signum larger than in other species, bearing dense and long spines.

#### Material examined.

**Type material. *Type***: 1♀, Ceylon (NHMUK).

#### Other material examined.

**Thailand**: 1♂, Chiengma, on eyes of horse, 24.VI.1963, W.W.G. Buttiker, Pyralidae Brit. Mus. Slide No. 12700 (NHMUK). **India**: 1♂, Bombay, 21.2.[18]92, Pyralidae Brit. Mus. Slide No. 010315440 (NHMUK). **China. Fujian**: 1♂1♀, Mt. Tianzhushan, Xiamen, 21, 24.VII.2014, Yang Xiaofei leg., genitalia slide No. ZDD12030 (♀) (NKU); **Guangdong**: 5♂3♀, Longyuan Ecological Garden, Zhepu Village, Hengli, Huizhou, 23.26°N, 114.60°E, 6.X.2021, Zhang Dandan leg.; **Hainan**: 2♂, Shuiman Village, Wuzhishan, 18.88°N, 109.66°E, alt. 667 m, 14.V.2013, 6.IX.2013, Chen Xiaohua, Li Jinwei leg., genitalia slide No. CXH12189; 1♂, Bawangling Natural Reserve, 19.08°N, 109.12°E, alt. 169 m, 10.V.2013, Li Jinwei leg., genitalia slide No. CXH12187; 2♂3♀, Jianfengling, 1–3.VI.2010, Kang Li leg.; 1♀, Jianling Natural Reserve, alt. 143 m, 18.52°N, 110.16°E, 8.IX.2013, Xie Weicai leg.; 1♂, Jianfeng, Ledong, 18.70°N, 108.80°E, alt. 58 m, 28.IV.2019, Xiang Lanbin leg.; 1♀, Yinggeling Natural Reserve, 19.05°N, 109.50°E, alt. 954 m, 4.IX.2013, Chen Xiaohua leg., genitalia slide No. SYSU0247, molecular voucher No. LEP0021; 1♂, Qijiafang, Limushan Natural Reserve, alt. 681 m, 15.IV.2016, Wei Xueli leg.; 1♂, Yaxing Village, Nankai, Baisha, 19.02°N, 109.40°E, alt. 321 m, 20.VI.2015, Cong Peixin, Guan Wei, Hu Sha leg. (NKU); 2♂1♀, Songtao Reservoir, Lanyang, alt. 194 m, 16–17.IV.2016, Wei Xueli leg.; **Yunnan**: 2♂5♀, Nabang Village, Yingjiang, 24.75°N, 97.56°E, alt. 239 m, 27.V.2016, Duan Yongjiang leg., genitalia slide No. SYSU0926 (♂, molecular voucher No. LEP0163); 1♂2♀, Longmen Village, Mengla, 23.VII.2011, Li Jinwei leg., genitalia slide No. SYSU0237 (♂, molecular voucher No. LEP0035); 1♂3♀, Tuanshan Village, Liming, Ninger, alt. 1162 m, 29.IV.2020, Xiang Lanbin leg.

**Figures 13–21.**
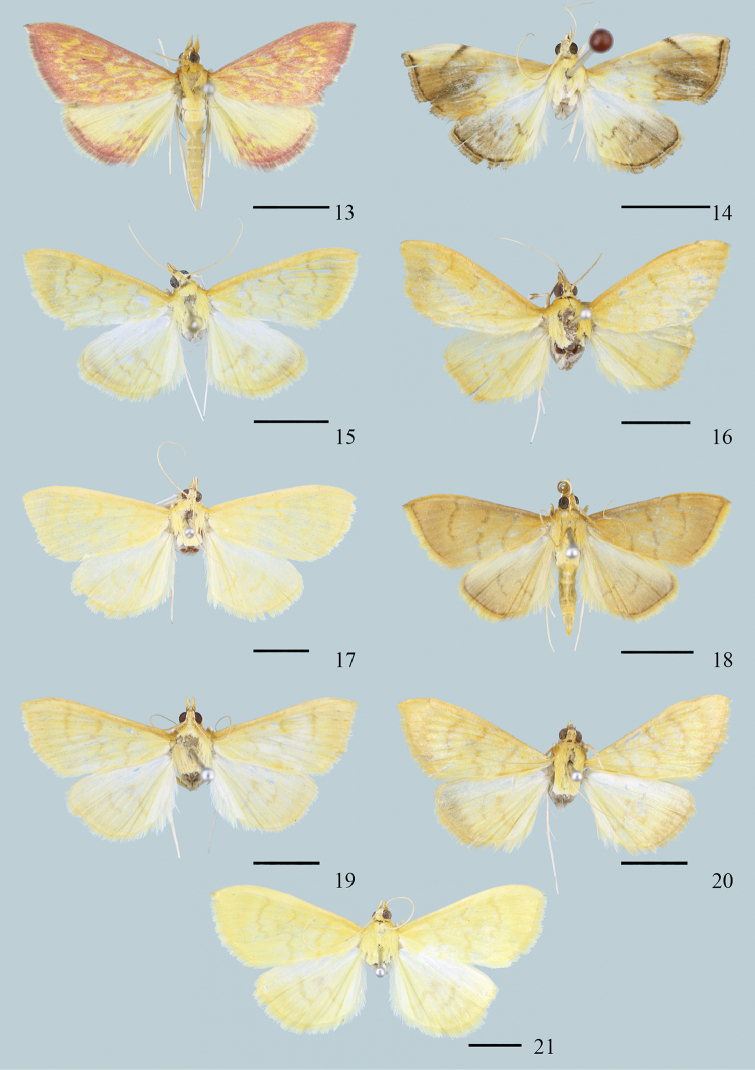
Adults of *Ecpyrrhorrhoe* spp. **13***E.rosisquama*, sp. nov., holotype, male (Yunnan) **14***E.exigistria*, sp. nov., holotype, male (Yunnan) **15***E.digitaliformis*, male (Guizhou) **16***E.brevis*, sp. nov., paratype, male (Guangdong) **17***E.puralis*, male (Hainan) **18***E.rubellalis*, male (Hainan) **19***E.longispinalis*, sp. nov., holotype, male (Hunan) **20***E.celatalis*, male (Yunnan) **21***E.biaculeiformis*, female (Anhui). Scale bars: 5.0 mm.

#### Distribution.

China (Fujian, Guangdong, Guangxi, Hainan, Yunnan), Sri Lanka, India, Thailand, Malaysia, Indonesia, Papua New Guinea, Australia.

#### Remarks.

The larvae of *Ecpyrrhorrhoedamastesalis* are leaf skeletonizers of teak (*Tectonagrandis*). Severe infestations, causing 90%–100% defoliation, has been recorded from Malaysia and Guangdong, China (Intachat 1998; Lin et al. 2018). The misidentification of *E.damastesalis* as *Ecpyrrhorrhoemachoeralis* (Walker, 1859), comb. nov. is common and has been verified in Java and Thailand (Intachat 1998) and Hainan, China (Wu et al. 1977; Wang 1980). We speculate that there are more misidentifications in the literature of this species as *E.machoeralis*.

### ﻿ Ecpyrrhorrhoe minnehaha

Taxon classificationAnimaliaLepidopteraCrambidae

(Pryer, 1877) comb. nov.

755A24B6-FE83-5E30-A5E3-B646DF76F7AF

Figs 6 , 7 , 24 , 39

Pyrausta minnehaha Pryer, 1877: 234.
Leucocraspeda auratalis Warren, 1895: 472. Syn. nov.
Pionea auratalis ab. obscura Caradja, 1935: 41.
Pionea schenklingi Strand, 1918: 79. Syn. nov.

#### Diagnosis.

This species can be differentiated from other species of the genus by its smaller forewing length (8.0–10.0 mm) and the yellow or rosy-red forewing usually bearing strongly contrasting spots and relatively smooth and slender lines (Figs 6, 7). In the male genitalia (Fig. 24), *E.minnehaha* is somewhat similar to *E.fimbriata*, but can be distinguished by the much more rounded ventral margin of valva, excurved sella densely bearing thick setae, pointed arms of juxta without tooth, and presence of a group of spines on the anellus. The female genitalia (Fig. 39) can be distinguished from congeners by the short cup-shaped antrum densely covered with minute spines.

**Figures 22–27.**
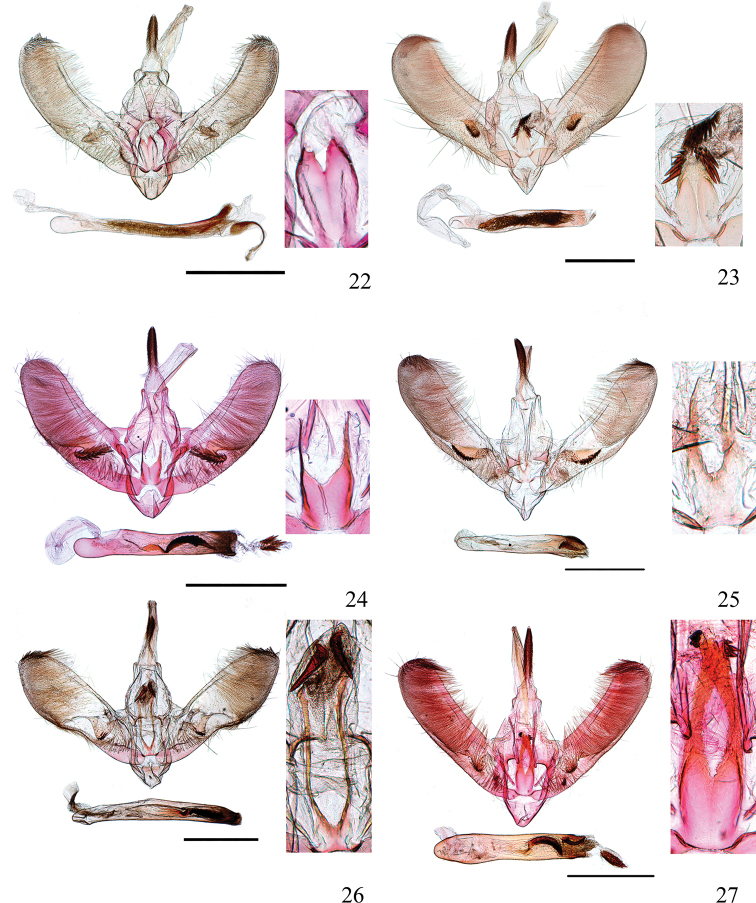
Male genitalia of *Ecpyrrhorrhoe* spp., with enlarged detail of juxta (right) **22***E.allochroa*, sp. nov., holotype, Yunnan (genitalia slide No. CXH12133) **23***E.damastesalis*, Yunnan (genitalia slide No. SYSU0237) **24***E.minnehaha*, Jiangxi (genitalia slide No. SYSU0271) **25***E.obliquata*, Hainan (genitalia slide No. SYSU0228) **26***E.rufipicta*, Hainan (genitalia slide No. ZDD12031) **27***E.fimbriata*, Yunnan (genitalia slide No. SYSU0281). Scale bars: 1.0 mm.

#### Redescription

(Figs 6, 7). **Head.** Frons yellow or yellowish brown with white lateral bands. Vertex pale yellow. Labial palpus yellow or yellowish brown, contrastingly white at base ventrally. Maxillary palpus yellow or yellowish brown, pale yellow terminally. **Thorax.** Dorsal side yellow or yellowish brown, ventral side white. **Wings.** Forewing length: 8.0–10.0 mm. Forewing yellowish brown, or rosy-red with posterior area straw yellow, markings blackish brown; antemedial line slightly arched from 1/4 of costa to 1/3 of posterior margin; orbicular stigma very small, dot-shaped, sometimes indistinct in rosy-red individuals; reniform stigma comma-shaped, nearly straight or weakly concave; postmedial line from 3/4 of costa to middle of CuA_1,_ bending to 1/3 of CuA_2_, then weakly curved to 2/3 of posterior margin; without distinct fuzzy patch posterolateral of cell indistinct; terminal line brown to dark brown; fringe with basal half brown to dark brown, distal half silver-white. Hindwing yellowish brown, with basal and subterminal area scattered with blackish brown scales, posterior area pale yellow; postmedial line from 2/3 of costa to 1/2 of CuA_1_, bending inward to 1/3 of CuA_2_, then weakly convex to 2/3 of posterior margin; the fuzzy patch posterolateral of cell blackish brown; terminal line and fringe as in forewing. **Abdomen.** Pale yellow dorsally, white ventrally.

#### Male genitalia

**(Fig. 24).** Uncus with basal 1/3 nearly triangular and naked. Valva with costa slightly concave and ventral margin curved, with apex rounded; sella excurved, thumb-shaped, densely bearing thick setae; sacculus with distal 3/5 inflated into a broad and nearly triangular protrusion. Juxta with base wide, distal half bifid into slender, straight, and widely separated arms; anellus with a cluster of short spines (attached to distal end of phallus in Fig. 24). Saccus rounded triangular. Phallus long and moderately thick; cornuti presented as a bunch of spines, with a large spine distally and connected with a lanceolate sclerite at base.

#### Female genitalia

**(Fig. 39).** Anterior apophysis ~ 2× length of posterior apophysis. Antrum cup-shaped, short and broad, strongly sclerotized, inner wall densely covered with minute spines; colliculum long, with two longitudinal ridges; ductus seminalis inserting to anterior end of colliculum and with a wide sclerite located opposite to colliculum; ductus bursae ~ 2× as long as length of corpus bursae, basal 2/3 bearing a slim sclerite. Corpus bursae oval; rhomboid signum narrow, with maximal length equal to half length of corpus bursae; second (posterior) signum small and somewhat curved, bearing short spines.

#### Material examined.

**Type material.***Pyraustaminnehaha*: ***Holotype*** ♂, [**China**]: Chekiang, 80.123 (NHMUK). *Leucocraspedaauratalis*: ***Holotype*** ♀, Japan (NHMUK). *Pioneaschenklingi*: ***Lectotype*** ♂, [**China**]: Kosempo, Formosa, X.1911, H. Sauter Coll., Gen. präp. Gaedike NR: 9669 (SDEI); ***Syntypes***: 1♀, [**China**]: Suisharyo, Formosa, X.[19]11, H. Sauter Coll., Gen. präp. Gaedike NR: 9670 (SDEI); 1♂, [**China**]: Suisharyo, Formosa, II.[19]12, H. Sauter Coll. (SDEI).

#### Other material examined.

**Material with yellowish brown forewing: China: Fujian**: 1♀, Mt. Mangdangshan, Maodi Village, 26.70°N, 118.08°E, alt. 812 m, 17.VIII.2016, Chen Kai, Duan Yongjiang leg.; **Guangdong**: 3♂, Niupoling, Yangchun, 18.VIII.2009, He Fengxia leg., genitalia slide LJW12053; 1♂, Mt. Danxiashan, Shaoguan, 25.04°N, 113.64°E, alt. 96 m, 7.VI.2012, Li Jinwei leg.; 1♂, Chebaling Nature Reserve, Shixing, 24.72°N, 114.26°E, alt. 496 m, 28.V.2017, Zhang Dandan leg., genitalia slide No. SYSU1013, molecular voucher No. LEP0217; 1♂, Dunzi Forest Farm, Huizhou, 10.IX.2015, Li Zhiqiang et al. leg.; **Guangxi**: 5♂, Gaozhai, Xing’an, 28.VIII.2011, Zhang Dandan, Li Jinwei leg., genitalia slide No. SYSU0225; 1♂, Gaozhai, Mt. Maoershan, Guilin, alt. 1100 m, 27.VII.2015, Liu Kaili, Zhao Jingxia leg.; **Guizhou**: 2♂, Baishao, Kuankuoshui Natural Reserve, alt. 800 m, 11.VIII.2010, Du Xicui leg.; **Yunnan**: 1♀, Yuanjiang, Yuxi, 23.97°N, 102.05°E, alt. 390 m, 16–17.VII.2019, Xiang Lanbin leg.; **Hubei**: 1♂, Wujiashan, Yingshan, 31.05°N, 115.47°E, alt. 880 m, 29.VI.2014, Chen Xiaohua, Pan Chang leg.; 1♂, Taohuachong, Mt. Dabieshan, alt. 590 m, 30.VI.2014, Xu Lijun leg. genitalia slide No. SYSU1043; **Hunan**: 1♂, Baimaoping, Chengbu, Shaoyang, 26.25°N, 110.37°E, alt. 550 m, 7–9.IX.2020, Jin Mengjie, Xiang Lanbin leg.; 2♂, Yangmeiao, Mt. Jiuwandashan, 25.19°N, 108.65°E, alt. 1183 m, 22.VII.2015, Chen Kai leg.; **Jiangxi**: 9♂, Shixi Village, Fengxin, 28.44°N, 114.54°E, alt. 506 m, 22.IX.2012, Li Jinwei, Yang Lijun leg., genitalia slide No. SYSU0227, molecular voucher No. LEP0033; 2♂, Guanyinyan, Jing’an, 29.03°N, 115.25°E, alt. 195 m, 20.VII.2014, Chenkai leg. genitalia slide No. SYSU0270, molecular voucher No. LEP0059; 1♂, Xiangzhou, Mt. Jinggangshan, 26.IV.2011, Yu Yali leg., genitalia slide No. SYSU0271, molecular voucher No. LEP0061; **Shanxi**: 9♂6♀, Miaoping, Manghe, 35.25°N, 112.46°E, alt. 557 m, 19–20.VIII.2018, Chen Kai, Xiang Lanbin leg.; **Shaanxi**: 1♀, Haopingsi, Yingtou, 34.05°N, 107.42°E, alt. 1251 m, 18.VII.2012, Li Jinwei leg., genitalia slide No. SYSU0272.

#### Material with rosy-red forewing:

**Japan**: 1♂, Nagasaki, May.1886, Leech, Pyralidae Brit. Mus. Slide No. 010315142 (NHMUK); **China: Hainan**: 1♀, Yinggeling, alt. 620 m, 18.IV.2010, Zhang Jing, Hu Bingbing leg. (abdomen missing).

#### Distribution.

China (Fujian, Guangdong, Guangxi, Guizhou, Hubei, Hunan, Jiangxi, Shaanxi, Shanghai, Shanxi, Taiwan, Yunnan, Zhejiang), Korea, Japan.

#### Remarks.

The type material of *Pyraustaminnehaha* Pryer, 1877 has rosy-red forewings bearing strongly contrasting spots and relatively smooth and slender lines, and the type series of *Pioneaschenklingi* Strand, 1918 has the same appearance. *Leucocraspedaauratalis* Warren, 1895 has a yellowish brown forewing, but bears the same markings, hindwings and fringe as *Pyraustaminnehaha* and *Pioneaschenklingi*. The genitalic characters indicate that these all belong to the same species. Although there is striking variation in this species, it is most easily identified by its appearance and its small body size. On the basis of currently available material, specimens with yellowish brown forewings are the commonest form in its range.

### ﻿ Ecpyrrhorrhoe obliquata

Taxon classificationAnimaliaLepidopteraCrambidae

(Moore, 1888) comb. nov.

974A77DA-373D-57BB-A33D-D9E2AE5E3FBA

Figs 8 , 25 , 40

Ebulea obliquata Moore, 1888: 224.
Leucocraspeda nissoralis Swinhoe, 1894: 145.

#### Diagnosis.

In appearance, *Ecpyrrhorrhoeobliquata* is similar to *E.exigistria* in the brown subterminal area of the wings, as well as in the oblique, dark brown streak on forewing which distinguish them from all other species of the genus. Genitalia characters readily distinguish *E.obliquata* from other members of the genus in the semi-circular sella with basal half of ventral margin bearing curved spines. It can be best distinguished from *E.exigistria* by the larger forewing length (forewing length: 10.0–12.0 mm), the more distinct and longer streak of the forewing, in the male genitalia (Fig. 25) by the shape of sella, and the weakly sclerotized arms of the bifid juxta, which is without teeth, and in the female genitalia (Fig. 40) by the shape of antrum.

#### Redescription

(Fig. 8). **Head.** Frons and vertex yellow, frons with lateral white bands. Labial palpus yellowish brown, contrastingly white at base ventrally. Maxillary palpus yellowish brown, white terminally. **Thorax.** Dorsal side yellow and ventral side white; tegula yellow with brown base. **Wings.** Forewing length: 10.0–12.0 mm. Forewing pale yellow, basal half of costal band and posterior half of subterminal area brown; stigmata and lines dark brown; antemedial line strongly oblique from 1/5 of costa to 1/2 of 1A, then dentated to 2/5 of posterior margin; orbicular stigma dot-shaped, sometimes indistinct; reniform stigma comma-shaped, thick and straight; an oblique, dark brown streak from posterior end of reniform stigma weakly curved and extended to tornus; postmedial line obliquely outwards from 3/5 of costa to 1/2 of M_1_, then dentated inwards to 2/3 of posterior margin; terminal line dark brown and intermittent; fringe with basal half yellow and blackish brown, distal half with anterior half yellow and posterior half dark brown. Hindwing pale yellow, subterminal area from dark brown to brown, gradually paler to tornus; postmedial line dark brown, slightly dentate from 2/3 of M_1_ to 1/2 of CuA_1_, then bending inwards to base of CuA_1_, finally undulated to 2/3 of posterior margin; terminal line and fringe as in forewing. **Abdomen.** Pale yellow dorsally, each segment with posterior margin whitish, black on distal end; white ventrally.

#### Male genitalia

**(Fig. 25).** Uncus with basal 1/3 nearly triangular and naked. Valva with costa nearly straight or concave and ventral margin curved, basal part weakly narrowed, remainder of even width, apex obtusely rounded; sella nearly semi-circular, bearing short and curved spines on distal half of ventral margins; sacculus with middle part inflated into a thumb-shaped protrusion. Juxta with base wide, distal 3/4 bifid into pointed arms. Saccus narrowly triangular. Phallus long and straight, with a bundle of short spines assembling into cone-shape at distal end.

#### Female genitalia

**(Fig. 40).** Anterior apophysis ~ 2× length of posterior apophysis. Antrum cup-shaped, with anterior 1/3 strongly sclerotized and covered with spinules on inner wall, posterior 1/3 abruptly broad and partly wrinkled; colliculum long; ductus seminalis connecting to anterior end of colliculum and with a short sclerite located opposite to colliculum; ductus bursae ~ 2.5–3× as long as diameter of corpus bursae, basal 1/3 bearing a slim sclerite. Corpus bursae globular; rhomboid signum with maximal length approximately 1/2 as long as diameter of corpus bursae; the second (posterior) signum nearly thumb-shaped bearing a wide base, sparsely covered with long spines.

#### Material examined.

**Type material**. *Leucocraspedanissoralis*: ***Lectotype*** (designated here) ♂, Kahsia Hs [Hills]. 94–66, Pyralidae Brit. Mus. Slide No. 10897 (NHMUK); ***Paralectotype* (designated here)**: 1♂, [**India**]: Cherre Punji (NHMUK).

#### Other material examined.

**China: Zhejiang**: 1♂, Mt. Jiulongshan, 5.VIII.2011, Fu Xiaobing leg.; **Jiangxi**: 2♂, Mt. Jiulianshan, Longnan, 24.58°N, 114.43°E, alt. 620 m, 26.IX.2016, 24.IX.2017, Chen Kai, Duan Yongjiang leg.; **Hunan**: 1♂, Xijialing, Mt. Shunhuangshan, Xinning, Shaoyang, 26.43°N, 111.01°E, alt. 1000 m, 6.IX.2020, Jin Mengjie, Xiang Lanbin leg.; 6♂2♀, Mt. Shunhuangshan, Xinning, Shaoyang, 26.40°N, 111°E, alt. 810 m, 4–6.IX.2020, Jin Mengjie, Xiang Lanbin leg.; 1♂, Dupangling National Natural Reserve, Dao County, Yongzhou, 25.48°N, 111.37°E, alt. 430 m, 29–30.VIII.2020, Jin Mengjie, Xiang Lanbin leg.; 1♀, Dupangling National Natural Reserve, Dao County, Yongzhou, 25.49°N, 111.39°E, alt. 350 m, 28–31.VIII.2020, Jin Mengjie, Xiang Lanbin leg.; 1♂, Qiaotoupu, Chengbu, Shaoyang, 26.25°N, 110.38°E, alt. 640 m, 8.IX.2020, Jin Mengjie, Xiang Lanbin leg.; **Guangdong**: 2♂1♀, Heishiding, Fengkai, 15.VI.2009, 9.X.2010, 2.VIII.2011, 9.VII.2017, 23.47°N, 111.90°E, alt. 214 m, Zhang Dandan et al. leg., genitalia slide No. LJW121067 (♂), SYSU0239 (♂), SYSU1236 (♀, molecular voucher no. LEP0297); 1♀, Yanshuitian, Fengkai, 6.IX.2011, Yang Lijun, Liao Junlei leg., genitalia slide No. SYSU0269; 2♂, Mt. Danxiashan, Shaoguan, 25.04°N, 113.64°E, alt. 96 m, 6–7.VI.2012, Li Jinwei leg., genitalia slide No. LJW12075, CXH12186; 1♀, Mt. Nankunshan, Huizhou, 16.VII.2003, Zhang Dandan, Li Zhiqiang leg., genitalia slide No. ZDD03057; 3♂2♀, Hongri Village, Mt. Nankunshan, Huizhou, 6–9.XI.2020, Jin Mengjie leg.; **Hainan**: 2♂, Bawangling Natural Reserve, 8.V.2011, Yang Lijun leg., genitalia slide No. LJW12101; 2♂, Yinggeling Natural Reserve, 19.05°N, 109.50°E, alt. 954 m, 4.IX.2013, Chen Xiaohua leg., genitalia slide No. CXH12212, SYSU0228 (molecular voucher No. LEP0034); 2♂, Hongkan, Yinggeling Natural Reserve, 19.08°N, 109.50°E, alt. 508 m, 15–16.VI.2015, Cong Peixin, Guan Wei, Hu Sha leg. (NKU); 1♂, Limushan Forest Park, 19.17°N, 109.73°E, alt. 607 m, 25.VII.2014, Cong Peixin, Liu Linjie, Husha leg. (NKU); 1♂, Wuzhishan Forest Park, 18.88°N, 109.67°E, alt. 766 m, 9.I.2016, Teng Kaijian, Bai Xia, Chen Mengting leg. (NKU); **Guangxi**: 1♂, Nonggang, Longzhou, 22.47°N, 106.96°E, alt. 271 m, 19.IV.2012, Li Jinwei leg., genitalia slide No. CXH12185; 1♂, Hekou, Dayaoshan Natural Reserve, Jinxiu, 24.14°N, 110.09°E, alt. 823 m, 20.VII.2015, Qi Mujie, Zhao Shengnan leg. (NKU); 2♂, Mt. Jiuwanshan, Hechi, alt. 1600 m, 23.VII.2015, Wang Jiping leg.; 1♂, Technology Building, Huaping Natural Reserve, Guilin, 25.63°N, 109.91°E, alt. 760 m, 10–12.IX.2020, Jin Mengjie, Xiang Lanbin leg.; **Yunnan**: 1♂, Taiyanghe Natural Reserve, alt. 1450 m, 23.VIII.2014, Zhang Zhenguo leg. (NKU); 1♂, Liaowangtai, Taiyanghe Forest Park, Pu’er, 22.60°N, 101.11°E, alt. 1626 m, 8.VII.2013, LiuShurong, Wang Yuqi, Teng Kaijian leg. (NKU); **Sichuan**: 1♂, Nuoshuihe Natural Reserve, Tongjiang, alt. 700 m, 5.VII.2013, He Guiqing, Xu Lijun leg.; **Chongqing**: 1♂, Daheba, Mt. Jinfoshan, alt. 800–850 m, 15.VII.2010, Du Xicui, Song Lifang leg.; 1♂, Tudiyan, Mt. Simianshan, alt. 1200 m, 9.VIII.2011, He Guiqing, Song Lifang leg.; **Tibet**: 1♂, Air-raid shelter, Beibeng, Medog, 29.24°N, 95.17°E, alt. 750 m, 31.VII.2018, Qi Mujie leg. (NKU); 1♂, Gelin, Beibeng, Medog, 29.25°N, 95.19°E, alt. 1063 m, 29.VII.2018, Qi Mujie leg. (NKU); 1♂, Yadong, Medog, 29.33°N, 95.34°E, alt. 833 m, 2.VIII.2018, Qi Mujie leg. (NKU).

#### Distribution.

China (Zhejiang, Jiangxi, Hunan, Guangdong, Hainan, Guangxi, Yunnan, Sichuan, Chongqing, Tibet), Burma, India, Sri Lanka.

### ﻿ Ecpyrrhorrhoe rufipicta

Taxon classificationAnimaliaLepidopteraCrambidae

(Butler, 1880) comb. nov.

F117B717-7D1F-5C5E-85D0-88C2ACC00451

Figs 9 , 26 , 41

Asopia rufipicta Butler, 1880: 682.
Paliga rubicundalis Warren, 1896: 96. Syn. nov.

#### Diagnosis.

Within the genus, *E.rufipicta* resembles *E.fimbriata* (Moore, 1886) in having nearly the same forewing length, yellow wings bearing brown markings and an almost indistinct, brown subterminal band (Fig. 9). However, it can be differentiated from *E.fimbriata* by more dentated and relatively thick postmedial line on both wings, in the male genitalia (Fig. 26) by the nearly oval valva, the thumb-shaped dorsal sella, the triangular ventral sella bearing several spines, the longer and strongly sclerotized arms of the juxta, and two long and pointed spines located on anellus.

#### Redescription

(Fig. 9). **Head.** Frons yellow, with lateral white bands. Vertex pale yellow. Labial palpus yellow, contrastingly white at base ventrally. Maxillary palpus yellow, white terminally. **Thorax.** Dorsal side yellow, and ventral side white; tegula yellow, with base brown. **Wings.** Forewing length: 9.0–12.0 mm. Wings yellow, with brown markings. Forewing with costal base brown; antemedial line dentated from 1/4 of costa slightly arched to 1/3 of posterior margin; orbicular stigma oblate; reniform stigma comma-shaped and thick; postmedial line dentated from 3/4 of costa to 2/5 of CuA_2_, then deeply dentated to 2/3 of posterior margin; subterminal band indistinct, with inner margin crenulated; fringe pale yellow. Hindwing with postmedial line brown, slightly dentated from 2/3 of M_1_ arched to 1/2 of CuA_1_, bending inward to basal 1/3 of CuA_1_, then dentated to near tornus; subterminal line and fringe as in forewing. **Abdomen.** Pale yellow dorsally, gradually brown to distal part, white ventrally.

#### Male genitalia

**(Fig. 26).** Uncus with basal 1/2 nearly triangular and naked. Valva with costa slightly convex and ventral margin curved, with basal part narrowed, remainder nearly oval, apex rounded; sella nearly triangular, bearing short spines on distal and inner margins, with a curved, finger-shaped dorsal projection; sacculus with middle part inflated into a triangular and setose protrusion. Juxta with base wide, distal 4/5 forming slender, long, and sclerotized arms dentate apically; anellus with two long and pointed spines (connected with distal arms of juxta in Fig. 26). Saccus rounded triangular. Phallus long and straight, cornuti presented as a narrow sclerite with dense and short spines.

#### Female genitalia

**(Fig. 41).** Anterior apophysis ~ 2.5× length of posterior apophysis. Antrum cylindrical, tuberculate laterally on anterior end, strongly sclerotized and covered with spinules on inner wall; colliculum long and broad, narrower medially; ductus seminalis connecting to anterior end of colliculum and with a wide sclerite located opposite to colliculum; ductus bursae ~ 2.5–3× as long as diameter of corpus bursae, basal 2/5 bearing a slim sclerite. Corpus bursae globular; rhomboid signum with maximal length > 1/2 of diameter of corpus bursae; second (posterior) signum composed of a pair of round sclerites bearing dense and long spines.

#### Material examined.

**Type material.***Asopiarufipicta*: **Type**: 1♀, [**China**:] Formosa, Pyralidae Brit. Mus. Slide No. 8682 (NHMUK). *Paligarubicundalis*: **Type**: 1♂, [**India**]: Khasis [Khasia] Nat. Coll., Pyralidae Brit. Mus. Slide No. 8685 (NHMUK).

#### Other material examined.

[**India**]: 1♂, Khasis [Khasia] Nat. Coll., NHMUK slide No. 010315123 (NHMUK); 1♂, Assam, NHMUK slide No. 010315163 (NHMUK); **Phlippines**: 1♂, Mt. Makiling, Luzon, Baker, 1917–79, Pyralidae Brit. Mus. Slide No. 19893 (NHMUK); **China: Guangxi**: 1♂, Miaozhai, Mt. Jinzhongshan, alt. 1450 m, 31.VII.2014, Wei Xueli, Ran Chao leg., genitalia slide No. SYSU1509; **Hainan**: 1♂, Baodao Village, Jiaxi Natural Reserve, 18.09°N, 109.05°E, alt. 149 m, 11.IX.2013, Xie Weicai leg., genitalia slide No. SYSU0645, molecular voucher No. LEP0038; 1♀, Jianling Natural Reserve, 18.87°N, 110.27°E, alt. 143 m, 8.IX.2013, Chen Xiaohua leg., genitalia slide No. SYSU0278, molecular voucher No. LEP0107; 1♂, Hongxin Village, Yuanmen, Baisha, 19.07°N, 109.52°E, alt. 460 m, 29.VI.2014, Cong Peixin, Liu Linjie, Hu Sha leg., genitalia slide No. ZDD12031, molecular voucher no. LEP0062 (NKU); 1♀, Hongkan, Yinggeling Natural Reserve, 19.08°N, 109.50°E, alt. 508 m, 15.VI.2015, Cong Peixin, Guan Wei, Hu Sha leg., genitalia slide No. SYSU0341 (NKU).

#### Distribution.

China (Guangxi, Hainan, Taiwan), India, Philippines.

#### Remarks.

Based on the substantial morphological similarity in the male genitalia between the types of *Asopiarufipicta* Butler, 1880 and *Paligarubicundalis* Warren, 1896, *Paligarubicundalis* is considered as a junior synonym of *E.rufipicta* (Butler).

### ﻿ Ecpyrrhorrhoe fimbriata

Taxon classificationAnimaliaLepidopteraCrambidae

(Moore, 1886) comb. nov.

6FF5ABAC-4DBA-55A3-A5D6-8299DE1E05CC

Figs 10 , 27

Ebulea fimbriata Moore, 1886: 346.
Ecpyrrhorrhoe angustivalvaris Gao, Zhang & Wang, 2013: 314. Syn. nov.

#### Diagnosis.

Forewing length: 9.0–11.0 mm. *Ecpyrrhorrhoefimbriata* is similar to *E.rubiginalis* both in appearance and in the male genitalia, but it can be differentiated from it by the relatively smooth and slender postmedial line on both wings (Fig. 10), in the male genitalia (Fig. 27), by the even width of the valva, dorsal projection of sella absent and the setose, thumb-shaped sella, the weakly sclerotized arms of the juxta with several short spines at apex, and the cluster of spines on the anellus (attached to distal end of phallus in Fig. 27).

#### Material examined.

**Type material.***Ebuleafimbriata*: ***Holotype*** ♂, Ceylon, Pyralidae Brit. Mus. Slide No. 8684 (NHMUK); ***Paratype***: 1♀, same data as holotype (NHMUK). *Ecpyrrhorrhoeangustivalvaris*: ***Holotype*** ♂, **China: Guizhou**: Dahe Dam, 28.33° N, 108.29°E, alt. 430 m, 6.VI.2007, Du Xicui leg., genitalia slide No. GQ11081 (NKU).

#### Other material examined.

**China: Guangxi**: 1♂, Nonggang, Longzhou, 22.47°N, 106.96°E, alt. 271 m, 19.VI.2012, Li Jinwei leg., genitalia slide No. LJW12065, molecular voucher No. LEP0039; 1♂, Shaoping Forestry Station, Pingxiang, alt. 280 m, 31.III.2012, Yang Xiaofei leg., genitalia slide No. CXH12139, molecular voucher No. LEP0099; 1♂, Huaping National Natural Reserve, Guilin, 25.63°N, 109.91°E, alt. 520 m, 11–12.IX.2020, Jin Mengjie, Xiang Lanbin leg., genitalia slide No. SYSU1507; **Guizhou**: 1♂, Fade Bridge, Shunchang, Shuicheng, 26.24°N, 104.85°E, alt. 857 m, 29.IV–3.V.2019, Liu Qingming leg., genitalia slide No. SYSU1506; **Yunnan**: 5♂, Baihualing, Baoshan, 11–13.VIII.2007, Zhang Dandan leg., genitalia slide No. CXH112169 (molecular voucher No. LEP0098), SYSU0115, SYSU0281 (molecular voucher No. LEP0111).

#### Distribution.

China (Guangxi, Guizhou, Yunnan), Sri Lanka.

#### Remarks.

After examination of the male genitalia of the holotypes of *Ebuleafimbriata* Moore, 1886 and *Ecpyrrhorrhoeangustivalvaris* Gao, Zhang & Wang, 2013, we conclude that they are the same species, sharing the same sella, juxta and phallus, even though the valva of the holotype of *E.angustivalvaris* is slightly narrower.

### ﻿ Ecpyrrhorrhoe rubiginalis

Taxon classificationAnimaliaLepidopteraCrambidae

(Hübner, 1796)

FD3CC115-25DD-56BA-A52B-81086E6B84F3

Figs 11 , 28 , 42

Pyralis rubiginalis Hübner, 1796: 22.
Pyrausta pygmaealis South, 1901: 505. Syn. nov.
Pionea rubiginalis delimbalis Schawerda, 1913: 170.
Pionea rubiginalis f. denigratalis Hartig & Amsel, 1952[1951]: 62.
Perinephela rubiginalis microlimbalis Amsel, 1959: 25.
Ecpyrrhorrhoe multispinalis Gao, Zhang & Wang, 2013: 312. Syn. nov.

#### Diagnosis.

**Figures 28–33.**
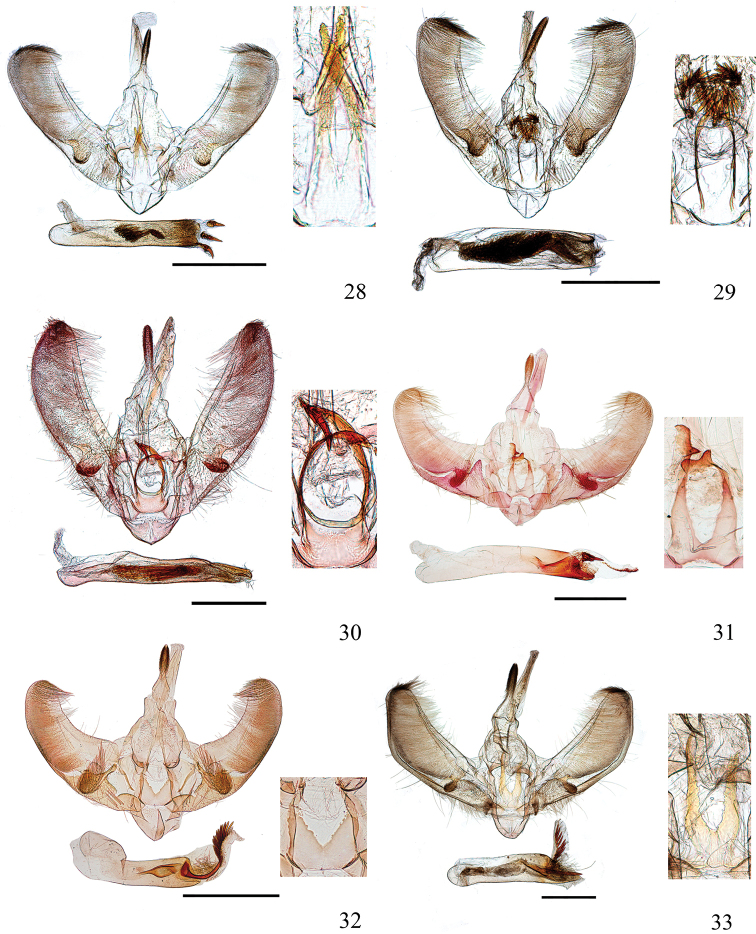
Male genitalia of *Ecpyrrhorrhoe* spp., with enlarged detail of juxta (right) **28***E.rubiginalis*, Shanxi (genitalia slide No. SYSU0245) **29***E.rosisquama* sp. nov., holotype, Yunnan (genitalia slide No. SYSU0246) **30***E.exigistria* sp. nov., holotype, Yunnan (genitalia slide No. ZDD12044) **31***E.digitaliformis*, Zhejiang (genitalia slide No. CXH12193) **32***E.brevis* sp. nov., holotype, Guangdong (genitalia slide No. CXH12182) **33***E.puralis*, Guangxi (genitalia slide No. SYSU0254). Scale bars: 1.0 mm.

Forewing length: 9.0–12.0 mm. In appearance, *Ecpyrrhorrhoerubiginalis* is similar to *E.fimbriata*, but the coloration of the wings and markings of *E.rubiginalis* is darker, and the patch in the hindwing is larger (Fig. 11). In the male genitalia (Fig. 28), the phallus of *E.rubiginalis* has a cluster of interlaced spicules and an oval sclerite bearing spines on the vesica, with three separate spines on the anellus (attached to distal end of phallus in Fig. 28).

#### Material examined.

**Type material.***Pyraustapygmaealis*: ***Lectotype*** (designated here) ♀, [**Chinia: Hubei**]: Ichang, Mrs Pratt Coll., June 1888, Pyralidae Brit. Mus. Slide No. 8681 (NHMUK). *Ecpyrrhorrhoemultispinalis*: ***Holotype*** ♂, **China: Tianjin**: Qilihai, 39.17°N, 117.34°E, 9.IX.2001, You Ping leg., genitalia slide No. GQ11075 (NKU).

#### Other material examined.

**China: Fujian**: 1♂, Chishui Station, Mt. Daiyunshan, 25.64°N, 118.14°E, alt. 1015 m, 22.V.2012, Li Jinwei leg., genitalia slide No. CXH12159; **Hainan**: 1♂, Yaxing Village, Nankai, Baisha, 19.02°N, 109.40°E, alt. 321 m, 20.VI.2015, Cong Peixin, Guan Wei, Hu Sha leg. (NKU); **Hebei**: 1♂ (abdomen missing), Pianchen Forestry Station, 36.44°N, 113.39°E, alt. 1109 m, 31.VII.2013, Liu Xiaolin leg.; **Heilongjiang**: 5♂1♀, Jiagedaqi, 13–14.VII.2012, Zhang Dandan, Yang Lijun leg., genitalia slide No. SYSU0238 (♂), CXH12176 (♂), CXH12177 (♂), CXH12179 (♂), CXH12264 (♀); 1♂, Dailing, Yichun, 20.VII.2012, Zhang Dandan, Yang Lijun leg., genitalia slide No. CXH12181; 1♂, Liangshui, Yichun, 19.VII.2012, Chen Xiaohua, Yang Lijun leg.; **Hubei**: 1♂, Huangbaoping Village, Hongping, Shennongjia, 31.61°N, 110.30°E, 24–25.VI.2019, 1530 m, Xiang Lanbin leg.; **Hunan**: 1♀, Yueyan Forestry Station, Dupangling National Natural Reserve, Dao County, Yongzhou, 25.49°N, 111.39°E, alt. 400 m, 28–31.VIII.2020, Jin Mengjie, Xiang Lanbin leg., genitalia slide No. SYSU1508, molecular voucher No. LEP0439; **Inner Mongolia**: 1♂, Linjiang Village, Erguna, 51.41°N, 119.97°E, alt. 475 m, 8.VIII.2019, Xiang Lanbin leg.; **Jiangxi**: 1♂, Xiaoxidong, Mt. Jinggangshan, 1.VII.2011, Yang Lijun leg.; 1♀, Mt. Jinggangshan, 2.X.2010, Chen Haidong, Xie Weicai leg., genitalia slide No. SYSU0215; 2♂, Mt. Guanggushan, Wuzhifeng, Shangyou, 25.92°N, 114.05°E, alt. 846 m, 22.VI.2015, Chen Kai leg., genitalia slide No. SYSU0207, SYSU0263; 1♂, Mt. Guanggushan, Shangyou, 25.92°N, 114.05°E, alt. 183 m, 20.IX.2016, Chen Kai, Duan Yongjiang leg.; 1♂, Qianmo Village, Nanfengmian Natural Reserve, Suichuan, 26.28°N, 114.06°E, alt. 816 m, 19.VI.2015, Chen Kai leg.; 1♂, Qianmo Village, Nanfengmian Natural Reserve, Suichuan, 26.29°N, 114.06°E, alt. 820 m, 19.IX.2017, Chen Kai leg.; 1♂1♀, Mt. Wugongshan, Luxi, 27.48°N, 114.15°E, alt. 500 m, 23.IX.2016, Chen Kai, Duan Yongjiang leg., genitalia slide No. SYSU0280 (♀), molecular voucher No. LEP0109; **Jilin**: 2♂, Wanbao Village, Antu, 23.VII.2012, Yang Lijun, Chen Xiaohua leg., genitalia slide No. CXH12180; 1♂, Huashan, Linjiang, 25.VII.2012, Yang Lijun, Chen Xiaohua leg., genitalia slide No. CXH12119; 1♂, Duanmusong, Linjiang, 26.VII.2012, Yang Lijun, Chen Xiaohua leg., genitalia slide No. CXH12016; 1♂, Hongshi Village, Hujia, Jiutai, 8.VIII.2018, Zhang Dandan leg.; **Shanxi**: 2♂, Dahe Forestry Station, Yicheng, 35.46°N, 111.93°E, alt. 1212 m, 13–15.VIII.2018, Xiang Lanbin leg.; 1♂, Dahe Village, Yicheng, 35.27°N, 111.56°E, alt. 1204 m, 28.VII.2013, Liu Xiaolin leg., genitalia slide No. SYSU0245, molecular voucher No. LEP0019; 1♂, Shangchuan Village, Qinshui, 35.28°N, 112.01°E, alt. 1619 m, 25.VII.2013, Liuxiaolin leg.; 3♂, Xiachuan Village, Qinshui, 35.44°N, 112.02°E, alt. 1514 m, 16–18.VIII.2018, Chen Kai, Xiang Lanbin leg.; 2♂1♀, Miaoping, Manghe, 35.25°N, 112.46°E, alt. 557 m, 19–20.VIII.2018, Chen Kai, Xiang Lanbin leg.; **Xinjiang**: 1♂, Nalati, Xinyuan, 43.31°N, 84.03°E, alt. 1389 m, 24.VII.2013, Li Jinwei leg., genitalia slide No. CXH12055; 1♂1♀, Baishahu, Habahe, Altay, 48.37°N, 85.74°E, alt. 553 m, 10.VII.2017, Chen Kai, Duan Yongjiang leg.; 5♂3♀, Yeguolin, Xinyuan, 43.38°N, 83.56°E, alt. 1282 m, 1.VII.2017, Chen Kai, Duan Yongjiang leg.

#### Distribution.

China (Beijing, Fujian, Hainan, Hebei, Heilongjiang, Henan, Hubei, Hunan, Inner Mongolia, Jiangxi, Jilin, Shaanxi, Shanxi, Tianjin, Xinjiang), Russia, Japan, Iran, Europe.

### ﻿ Ecpyrrhorrhoe machoeralis

Taxon classificationAnimaliaLepidopteraCrambidae

(Walker, 1859) comb. nov.

447EF5A0-707C-5C48-B635-A1C25416785F

Figs 12 , 43

Scopula machoeralis Walker, 1859: 1013.
Eutectona machaeralis [sic]: Wang & Sung, 1980: 305.

#### Diagnosis.

Forewing length: 8.0–10.0 mm. *Ecpyrrhorrhoemachoeralis* can be differentiated from congeners by the antrum with anterior half narrower than posterior half.

#### Female genitalia

(Fig. 43). Anterior apophysis ~ 1.5× length of posterior apophysis. Antrum long and tubular, with anterior half broad and slightly wrinkled, posterior half slightly narrow and as wide as colliculum; colliculum moderately long, somewhat difficult to differentiate from antrum; ductus seminalis connecting to anterior end of colliculum and with a weak sclerite located opposite to colliculum; ductus bursae ~ 2–2.5× as long as diameter of corpus bursae, basal 2/5 bearing a slim sclerite. Corpus bursae globular; rhombic signum with maximal length < ~ 1/2 length of diameter of corpus bursae; second (posterior) signum curved, with both ends bearing sparse and long spines.

#### Material examined.

**Type material. *Type***: 1♀, Ceylon, genitalia slide No. 8680 (NHMUK).

#### Distribution.

Sri Lanka.

#### Remarks.

Based on the examined material, this species only occurs in Sri Lanka, from where the type was described (Walker 1859). However, because of the misidentifications as *Ecpyrrhorrhoemachoeralis* of *E.damastesalis*, records of the former are widely present in the literature (Wu et al. 1977, Intachat 1998, Lin et al. 2018, Wang 1980). The reported biology of *E.machoeralis* needs to be confirmed.

The damaged female type material of *E.machoeralis* and lacks the original wing pattern because of its lack of scales, and the genital dissection is of low quality, which makes the identification and diagnosis of *E.machoeralis* difficult.

### ﻿ Ecpyrrhorrhoe rosisquama

Taxon classificationAnimaliaLepidopteraCrambidae

Xiang & Zhang sp. nov.

D8C2B8A4-72DC-5349-B1C7-7C77335D94FA

http://zoobank.org/1D83FACD-6109-46DB-8735-27A387D7974B

Figs 13 , 29 , 44

#### Diagnosis.

This species differs from all other species of the genus by the yellow forewing bearing rosy markings and mottled covering of rosy scales, the rosy markings on tornal area of the hindwing (Fig. 13), in the male genitalia (Fig. 29) by the longer and more strongly sclerotized arms on the juxta, with two groups of short and pointed spines on the anellus, in the female genitalia (Fig. 44) by the smaller and less developed antrum, and the smaller corpus bursae.

#### Description

(Fig. 13). **Head.** Frons and vertex yellow, frons with lateral white bands. Labial palpus yellow or orange, contrastingly white at base ventrally. Maxillary palpus yellow, pale terminally. Frons, vertex, labial and maxillary palpi sometimes mixed with rosy scales. **Thorax.** Dorsal side and tegula yellow, mixed with rosy scales; ventral side white. **Wings.** Forewing length: 10.0–12.0 mm. Forewing with termen weakly oblique; ground color yellow, with mottled covering of rosy scales forming indistinct markings except the totally rosy costa; antemedial line blurred, from 1/5 of costa oblique to 1/3 of 1A, then dentate to 2/5 of posterior margin; orbicular stigma nearly square; reniform stigma comma-shaped, thick and concave; postmedial line blurred, arched from 3/4 of costa to base of CuA_2_, connected with oval-shaped patch posterolateral of cell, and finally dentated to 2/3 of posterior margin; subterminal band with anterior part broad, inner margin serrated; fringe with basal half dark rosy and distal half pale yellow. Hindwing pale yellow; postmedial and subterminal lines rosy and serrated, but only with posterior part distinct; terminal line sometimes rosy; fringe as in forewing, with apex and tornus pale yellow. Underside: costal and terminal areas grey; reniform stigma and patch posterolateral of cell grey and distinct; postmedial line grey and faint. **Abdomen.** Yellowish to yellowish brown dorsally, the first two segments whitish, posterior margin of each segment paler. Dirty white ventrally.

#### Male genitalia

**(Fig. 29).** Uncus with basal 1/4 nearly triangular and naked. Valva slightly curved, of almost even width, apex obtusely rounded; sella thick and setose, thumb-shaped, bearing short spines on distal margin, extending ventrad; sacculus with distal 3/4 inflated into a broad and round protrusion. Juxta with base wide, distal 4/5 bifid into slender, long, and sclerotized arms; anellus with two groups of short and pointed spines (connected with distal arms of juxta in Fig. 29). Saccus rounded triangular. Phallus rather stout and straight, with interlaced spicules on vesical and cornuti present as a sclerite with dense and long spines.

#### Female genitalia

**(Fig. 44).** Anterior apophysis ~ 2× the length of posterior apophysis. Antrum cup-shaped, weakly sclerotized, slightly wrinkled medially; colliculum moderately long and broad; ductus seminalis inserting to anterior end of colliculum and with a wide sclerite and a nearly semi-circular, strong sclerotized sclerite located opposite colliculum; ductus bursae ~ 3–4× diameter of corpus bursae, basal half bearing a slim sclerite. Corpus bursae globular; rhombic signum with maximal length < 1/2 of diameter of corpus bursae; second (posterior) signum composed of a pair of narrow and pointed sclerites without spines.

#### Material examined.

**Type material. *Holotype*** ♂, **China: Yunnan**: Baihualing Natural Reserve, Baoshan, 24.30°N, 98.80°E, alt. 1535 m, 20.IV.2015, Chen Kai, Duan Yongjiang leg., genitalia slide No. SYSU0246, molecular voucher No. LEP0020 (SYSBM). ***Paratypes*: Guangxi**: 1♀, Songshuping, Mt. Jinzhongshan, alt. 940 m, 28.VII.2014, Wei Xueli, Ran Chao leg.; **Yunnan**: 5♂7♀, Baihualing Natural Reserve, Baoshan, alt. 1520 m, 11, 13.VIII.2007, Zhang Dandan leg., genitalia slide No. SYSU0209 (♂), SYSU0229 (♂), SYSU0262 (♀); 1♀, Baihualing Natural Reserve, Baoshan, 25.30°N, 98.80°E, alt. 1473 m, 7.VIII.2014, Teng Kaijian, Liu Shurong, Rong Hua leg. (NKU); 1♀, Hanlongzhai, Baihualing Natural Reserve, Baoshan, 25.31°N, 98.80°E, alt. 1616 m, 11.V.2021, Jin Mengjie, Guo Muyu, Fu Haiyun leg.; 1♂, Taizhong Village, Jingdong, 24.51°N, 100.94°E, alt. 1395 m, 14.IV.2015, Chen Kai, Duan Yongjiang leg.; 2♀, Tuanshan Village, Liming, Ninger, alt. 1162 m, 29.IV.2020, Xiang Lanbin leg.

#### Distribution.

China (Guangxi, Yunnan).

#### Etymology.

The specific name is derived from the Latin *rose*- (= rosy) and *squama* (= scales), referring to rosy scales on wings.

### ﻿ Ecpyrrhorrhoe exigistria

Taxon classificationAnimaliaLepidopteraCrambidae

Zhang & Xiang sp. nov.

A0C9121E-1B58-5B71-A024-655724CF5FEA

http://zoobank.org/F4EE0BEE-14BF-4D43-A4EA-54AE5F66F4CA

Figs 14 , 30 , 45

#### Diagnosis.

This species is similar to *E.obliquata* but can be best distinguished from it by the smaller size (forewing length: 7.0–9.0 mm), the pale brown reniform stigma and the indistinct and short streak of the forewing (Fig. 14), in the male genitalia (Fig. 30) by the excurved, finger-shaped and setose sella, the strongly sclerotized arms of the juxta, and anellus with a long spine and one or two short spines and in the female genitalia (Fig. 45) by the mostly tubular antrum.

#### Description

(Fig. 14). **Head.** Frons and vertex yellow, frons with lateral white bands. Labial palpus yellowish brown, contrastingly white at base ventrally. Maxillary palpus yellowish brown, white terminally. **Thorax.** Dorsally yellow, ventrally white; tegula yellow with brown base. **Wings.** Forewing length: 7.0–9.0 mm. Forewing pale yellow, posterior half of subterminal area brown; antemedial line dark brown, almost straight from 1/5 of costa to 2/5 posterior margin, slightly dentate; reniform stigma comma-shaped, brown; an oblique, dark brown streak from posterior end of reniform stigma, fuzzy, extended to tornus; postmedial line black brown, obliquely outwards from 3/5 of costa to 1/2 of M1, then dentated inwards to 2/3 of posterior margin; terminal line black brown; fringe with basal half brown, distal half dark brown. Hindwing pale yellow, subterminal area from dark brown to yellow, gradually paler to tornus; postmedial line black-brown, slightly dentate from 2/3 of M_1_ to 1/2 of CuA_1_, then bending inwards to base of CuA_1_, finally undulated to 2/3 of posterior margin; terminal line and fringe as in forewing. **Abdomen.** Pale yellow dorsally, black distally, white ventrally.

#### Male genitalia

(Fig. 30). Uncus with basal 2/5 nearly triangular and naked. Valva narrowly oval, costa straight or slightly concave and ventral margin curved, with apex rounded; sella excurved, thumb-shaped, thick, and setose, bearing thick setae on distal half; sacculus with distal 3/5 inflated into a broad and nearly triangular protrusion. Juxta with base wide, distal 3/4 bifid into slender, curved, sclerotized, and widely separated arms; anellus with a long spine and one or two short spines (attached to distal part of juxta in Fig. 30). Saccus rounded triangular. Phallus long and tapering, distal end spinulose and weakly sclerotized, with interlaced spicules cluster on vesica.

#### Female genitalia

(Fig. 45). Anterior apophysis ~ 1.5× length of posterior apophysis. Antrum long tubular and sclerotized, with anterior 1/3 broad, weakly sclerotized and wrinkled medially; colliculum long; ductus seminalis connecting to anterior end of colliculum and with a weak sclerite located opposite to colliculum; ductus bursae ~ 2–2.5× as long as diameter of corpus bursae, basal 1/3 bearing a slim sclerite. Corpus bursae globular; rhomboid signum with maximal length > 1/2 of diameter of corpus bursae; second (posterior) signum curved, oval, bearing sparse and long spines.

#### Material examined.

**Type material. *Holotype*** ♂, **China: Yunnan**: Wild Elephant Valley, Xishuangbanna, 22.17°N, 100.87°E, alt. 762 m, 12.VII.2015, Teng Kaijian, Bai Xia leg., genitalia slide No. ZDD12044, molecular voucher No. LEP0063 (SYSBM). ***Paratypes*. China: Guangxi**: 1♂, Lianhuashan, Mt. Dayaoshan, alt. 1250 m, 22.VII.2015, Liu Kaili, Zhao Jingxia leg., genitalia slide No. SYSU1009, molecular voucher No. LEP0211; 1♂, Mt. Shengtangshan, Jinxiu, 25.VIII.2011, Cheng Muchun leg., genitalia slide No. LJW12080; **Hainan**: 1♂, Jianfenling Natural Reserve, 18.75°N, 108.85°E, alt. 969 m, 12.V.2013, Li Jingwei leg., genitalia slide No. SYSU1247; 2♂, Diaoluoshan, Lingshui, 18.72°N, 109.87°E, alt. 942 m, 29–30.IV.2019, Xiang Lanbin leg., genitalia slide No. SYSU1514; **Jiangxi**: 1♀, Mt. Jiulianshan, Longnan, 24.58°N, 114.43°E, alt. 620 m, 26.IX.2016, Chen Kai, Duan Yongjiang leg., genitalia slide No. SYSU0276, molecular voucher no. LEP0100; 1♂, Xiagongtang, Mt. Jiulianshan, Ganzhou, 24.54°N, 114.46°E, alt. 600 m, 16.VIII.2020, Jin Mengjie leg., genitalia slide No. SYSU1513; **Tibet**: 1♂, Dexing Village, Medog, 29.32°N, 95.30°E, alt. 833 m, 18.VIII.2017, Qi Mujie, Yang Xiaofei leg. (NKU); **Yunnan**: 2♂, Wild Elephant Valley, Xishuangbanna, 22.17°N, 100.87°E, alt. 762 m, 18, 20.VII.2014, Teng Kaijian, Guan Wei, Wang Xiuchun, Liu Shurong leg. (NKU).

#### Distribution.

China (Guangxi, Hainan, Jiangxi, Tibet, Yunnan).

#### Etymology.

The specific name is derived from the Latin *exigu*- (= short) and *stria* (= streak), referring to the short streak on forewings.

### ﻿ Ecpyrrhorrhoe digitaliformis

Taxon classificationAnimaliaLepidopteraCrambidae

Zhang, Li & Wang, 2004

6F5894DC-7A3E-58AC-BF5B-29536D0828A1

Figs 15 , 31 , 46

Ecpyrrhorrhoe digitaliformis Zhang, Li & Wang, 2004: 318.

#### Diagnosis.

Forewing length: 9.0–14.0 mm. In appearance, *Ecpyrrhorrhoedigitaliformis* is indistinguishable from *E.celatalis* (Walker, 1859), but it can be distinguished from it in the male genitalia (Fig. 31) by the tapering and curved valva, the thumb-shaped, excurved and setose sella inflated distally, by the stout, sclerotized, finger-shaped dorsal protrusion of the sacculus, by the anellus with a string of minute spines and a cone-shaped group of large spines (attached to distal phallus in Fig. 31), the modified distal ends of the arms of the juxta, and the shape of cornuti; in the female genitalia (Fig. 46), by the antrum with a vertical wrinkled area in the middle.

This species is closely related to *E.brevis* based on molecular data, and similar in appearance and male genitalia, but can be differentiated by the slender and excurved sella, the thick protrusion of sacculus, and the slender arms of juxta bearing a tooth-shaped process, as well as the characters mentioned above.

#### Material examined.

***Holotype*** ♂, **China: Henan**: Xinyang, 32.06°N, 114.07°E, alt. 700 m, 13.VII.2013, Zhang Dandan leg., genitalia slide No. ZDD02107 (NKU). ***Paratypes*: China: Henan**: 1♀, same data as holotype, genitalia slide No. ZDD02115 (NKU); **Zhejiang**: 1♂1♀, Mt. Tianmushan, 30.26°N, 119.34°E, 16.VIII.1999, Li Houhun leg. (NKU).

#### Other material examined.

**China: Chongqing**: 2♂, Wuli, Qianjiang, alt. 870 m, 24.VII.2012, Zhang Jun, Xu Lijun leg., genitalia slide No. SYSU1528; 1♂1♀, Mt. Jinyinshan, Qianjiang, alt. 1100 m, 25.VII.2012, Zhang Jun, Xu Lijun leg., genitalia slide No. SYSU1551 (♂); 3♂2♀, Xiaonanhai, Qianjiang, alt. 370 m, 21.VII.2012, Zhang Jun, Xu Lijun leg., genitalia slide No. SYSU1550 (♂); **Guangdong**: 1♂, Niupoling, Yangchun, 18.VIII.2009, He Fengxia leg., genitalia slide No. HFX08237; 2♀, Dawuling, Xinyi, alt. 900 m, 7–14.VIII.2003, Zhang Dandan, Jian Yuening, Lin Meiying leg., genitalia slide No. ZDD003023, ZDD03072; **Guangxi**: 5♂2♀, Mt. Shengtangshan, Jinxiu, 25–26.VIII.2011, Yang Lijun, Cheng Muchun, Zhang Dandan leg., genitalia slide No. CXH12164(♂), CXH12175(♂), CXH12183(♂), CXH12214(♀), SYSU0274(♀, molecular voucher No. LEP0402); 1♂, Hekou, Dayaoshan Natural Reserve, Jinxiu, 24.14°N, 110.09°E, alt. 823 m, 20.VII.2015, Qin Mujie, Zhao Shengnan leg. (NKU); 1♀, Gaozhai Village, Xing’an, 28.VIII.2011, Li Jinwei leg., genitalia slide No. SYSU1522; 1♂, Anjiangping Natural Reserve, 25.56°N, 109.93°E, alt. 1751 m, 10.VII.2013, Chen Xiaohua leg., genitalia slide No. SYSU1527; **Guizhou**: 3♂, Maolan Natural Reserve, Libo, 25.25°N, 107.90°E, alt. 814 m, 25.VII.2015, Chen Kai leg., genitalia slide No. SYSU0217, SYSU0221, SYSU0051; 1♂, Taojiang, Leishan, 27.VIII.2012, Li Jinwei leg., genitalia slide No. CXH12160; **Hainan**: 2♂1♀, Yinggeling, 19.05°N, 109.50°E, alt. 954 m, 4.IX.2013, Chen Xiaohua, Xie Weicai leg., genitalia slide No. SYSU0224 (♂); **Hubei**: 2♂, Taohuachong, Mt. Dabieshan, 30.59°N, 116.19°E, alt. 661 m, 24.VI.2014, Chen Xiaohua, Pan Chang leg., genitalia slide No. SYSU0208, SYSU0241; 1♂, Qingtaiguan, Luotian, 31.11°N, 115.41°E, alt. 524 m, 2.VII.2014, Liu Zhenhua, Pan Chang leg., genitalia slide No. SYSU0214; **Hunan**: 1♀, Baiyun Reservoir, Baimaoping, Chengbu, Shaoyang, 26.27°N, 110.36°E, alt. 560 m, 7.IX.2020, Jin Mengjie, Xiang Lanbin leg., genitalia slide No. SYSU1565; 1♂, Zhupo Village, Huitong, 23.VIII.2012, Li Jinwei, Chen Xiaohua leg., genitalia slide No. CXH12198; 1♂, Yueyan Village, Dao County, 21.VIII.2012, Li Jinwei, Chen Xiaohua leg., genitalia slide No. CXH12197; **Jiangxi**: 1♂, Xiaoxidong, Mt. Jinggangshan, 1.VII.2011, Yang Lijun leg., genitalia slide No. SYSU0235; 1♀, Xiaoxidong, Mt. Jinggangshan, 2.VIII.2011, Li Jinwei leg.; 2♂4♀, Mt. Jiulianshan, Longnan, 24.58°N, 114.43°E, alt. 620 m, 26.IX.2016, 24.IX.2017, Chen Kai, Duan Yongjiang leg., genitalia slide No. SYSU1547; 1♂, Mt. Wugongshan, Luxi, 27.48°N, 114.15°E, alt. 500 m, 23.IX.2016, Chen Kai, Duan Yongjiang leg., genitalia slide No. SYSU1549; **Shaanxi**: 1♂, Yueba, Foping, 33.55°N, 107.82°E, alt. 1052 m, 1–3.VIII.2018, Liu Qingming, Xiang Lanbin leg., genitalia slide No. SYSU1543; 1♂, Longcaoping, Foping, 33.65°N, 107.97°E, alt. 1218 m, 4.VIII.2018, Liu Qingming, Xiang Lanbin leg., genitalia slide No. SYSU1544; **Yunnan**: 1♂1♀, Taiyanghe Reserve, alt. 1450 m, 15.VIII, 2.IX.2014, Zhang Zhenguo leg., genitalia slide No. ZDD12027(♂), ZDD12118(♀) (NKU); **Zhejiang**: 1♂, Mt. Tianmushan, Lin’an, 30.31°N, 119.44°E, alt. 295 m, 11.V.2012, Li Jinwei leg., genitalia slide No. CXH12193; 1♂2♀, Mt. Tianmushan, alt. 400 m, 25.VII.2011, Du Xicui leg., genitalia slide No. SYSU1529(♂).

#### Distribution.

China (Chongqing, Guangdong, Guangxi, Guizhou, Hainan, Henan, Hubei, Hunan, Jiangxi, Shaanxi, Yunnan, Zhejiang).

### ﻿ Ecpyrrhorrhoe brevis

Taxon classificationAnimaliaLepidopteraCrambidae

Zhang & Xiang sp. nov.

AC5A2121-4E8B-532A-9113-33A9DBDA2BE4

http://zoobank.org/595A86BB-2342-4A3D-89DD-701F073EEA44

Figs 16 , 32 , 47

#### Diagnosis.

*Ecpyrrhorrhoebrevis* can be distinguished from *E.digitaliformis* and *E.celatalis* in the male genitalia (Fig. 32) by the much more slender protrusion of the sacculus, the much thicker and straight sella, the broader arms of the juxta without a tooth-shaped process, a long, strongly sclerotized and hook-like cornutus present on the vesica, and a series of long spines standing on a long and curved base on the anellus; in the female genitalia (Fig. 47) by anterior end of the antrum bearing a lateral protrusion.

#### Description

(Fig. 16). **Head.** Frons yellow, with white lateral bands. Vertex pale yellow, sometimes whitish medially. Labial palpus dark yellow, contrastingly white at base ventrally. Maxillary palpus dark yellow, pale terminally. **Thorax.** Dorsal side dark yellow or yellowish brown, ventral side white. Legs white to pale yellow. **Wings.** Forewing length: 11.0–13.0 mm. Forewing with termen moderately oblique and apex somewhat pointed; bright yellow, markings yellowish brown; antemedial line arched from 1/4 of costa to 2/5 of posterior margin; orbicular stigma dot-shaped, sometimes indistinct; reniform stigma comma-shaped, slightly concave; postmedial line from 3/4 of costa, obliquely inward then arched to middle of CuA_1_, slightly dentate, then bending to 1/3 of CuA_2_, and finally undulated to 2/3 of posterior margin; fringe bright yellow. Hindwing bright yellow; postmedial line yellowish brown, slightly dentate and arched from 2/3 of M_1_ to 1/2 of CuA_1_ and bending inward along CuA_1_, then undulated to 2/3 of posterior margin; fringe as in forewing. **Abdomen.** Bright yellow dorsally, black on distal end; white ventrally.

#### Male genitalia

**(Fig. 32).** Uncus with basal half nearly triangular and naked. Valva curved and slowly tapering to rounded apex; sella thickly sclerotized, thumb-shaped, and densely setose; sacculus with distal 3/5 inflated into a broad protrusion bearing a slender finger-shaped process medially. Juxta with base wide, distal 4/5 bifid into stout and tapering, slightly curved and closely separated arms; anellus with a series of long spines standing on a long and curved base (attached to distal end of phallus in Fig. 32). Saccus broadly triangular. Phallus long and moderately stout, cornuti presented as a lancet-shaped sclerite connected with a hook-shaped, strong spine on apical end.

#### Female genitalia

**(Fig. 47).** Anterior apophysis ~ 2× length of posterior apophysis. Antrum shortly cup-shaped, anterior end of antrum bearing a lateral thumb-shaped protrusion; colliculum very short and broad; ductus seminalis connecting to anterior end of colliculum; ductus bursae length ~ 4–5× as long as diameter of corpus bursae, basal 1/3 bearing a slim sclerite. Corpus bursae globular; rhomboid signum with maximal length > diameter of corpus bursae; second (posterior) signum with both ends round, margin bearing sparse and long spines.

#### Material examined.

**Type material. *Holotype*** ♂, **China: Guangdong**: Heishiding, Fengkai, 5.X.2011, Tong Bo, Li Yun leg., genitalia slide No. CXH12182(♂) (SYSBM). ***Paratypes*: China: Guangdong**: 2♂1♀, same data as holotype, genitalia slide No. SYSU0234(♂), SYSU0236(♂), CXH12213(♀, molecular voucher No. LEP0398); 1♂, Heishiding, Fengkai, 15.VI.2009, Han Xiaolei leg., genitalia slide No. SYSU1532; 1♀, Heishiding, Fengkai, 25.V.2013, Chen Xiaohua leg., genitalia slide No. SYSU1533; 1♂, Yanshuitian, Fengkai, 6.X.2011, Tong Bo leg., genitalia slide No. SYSU0253; 1♂, Mt. Danxiashan, Shaoguan, 25.04°N, 113.64°E, alt. 96 m, 6.VI.2012, Li Jinwei leg., genitalia slide No. SYSU0212, molecular voucher No. LEP0036. **Guangxi**: 1♂, Yangmeiao, Mt. Jiuwandashan, 25.19°N, 108.65°E, alt. 1183 m, 22.VII.2015, Chen Kai leg., genitalia slide No. SYSU0268.

#### Distribution.

China (Guangdong, Guangxi).

#### Etymology.

The specific name is derived from the Latin *brevis* (= short), referring to the short arms of juxta in the male genitalia.

### ﻿ Ecpyrrhorrhoe puralis

Taxon classificationAnimaliaLepidopteraCrambidae

(South, 1901)

462D50CB-6EBA-5BBF-9939-7F5638AB02C1

Figs 17 , 33 , 48

Pionea puralis South, 1901: 493.

#### Diagnosis.

Forewing length: 11.0–14.0 mm. *Ecpyrrhorrhoepuralis* is almost indistinguishable from *E.longispinalis* and *E.biaculeiformis* in appearance, but can be distinguished in the male genitalia (Fig. 33) by the small and excurved sella, much larger juxta with distal 2/3 bifid, anellus with comb-shaped spines (attached to distal phallus end of in Fig. 33), and in the female genitalia (Fig. 48) by the antrum with two rounded sclerotized processes, and basal 2/3 of ductus bursae bearing a slim sclerite.

#### Material examined.

***Type***: 1♂, [**China: Hubei**:] Ichang, Mrs Pratt Coll., June 1888, Pyralidae Brit. Mus. Slide No. 8676 (NHMUK).

#### Other material examined.

**China: Guangdong**: 2♂4♀, Heishiding, Fengkai, 5.IX, 1.V, 5.X.2011, Tong Bo, Zhang Dandan, Li Yun, Yang Lijun, Cheng Muchun, Liao Junlei leg., genitalia slide No. CXH12170(♂), CXH12216(♀); **Guangxi**: 1♂1♀, Mt. Jinzhongshan, 24.67°N, 104.88°E, alt. 957 m, 18.VII.2013, Chen Xiaohua leg., genitalia slide No. SYSU0205(♀); 4♂2♀, Yangmeiao, Mt. Jiuwandashan, 25.19°N, 108.65°E, alt. 1183 m. 22.VII.2015, Chen Kai leg., genitalia slide No. SYSU0254(♂, molecular voucher No. LEP0161), SYSU0257(♀), SYSU0258(♂, molecular voucher No. LEP0399); **Hebei**: 1♂1♀, Piancheng Forestry Station, She County, 36.44°N, 113.39°E, alt. 1109 m, 31.VII.2013, Xie Weicai, Liu Xiaolin leg., genitalia slide No. SYSU1539(♂); **Hubei**: 1♀, Wujiashan, Yingshan, 31.05°N, 115.47°E, alt. 880 m, 29.VI.2014, Chen Xiaohua, Pan Chang leg., genitalia slide No. SYSU1540; 2♂, Qingtaiguan, Luotian, 31.11°N, 115.41°E, alt. 524 m, 2.VII.2014, Liu Zhenhua, Pan Chang leg., genitalia slide No. SYSU1518(♂); **Hunan**: 1♀, Jiashui, Taoyuandong, 26.59°N, 113.99°E, alt. 420 m, 19.V.2014, Chen Xiaohua leg., genitalia slide No. SYSU0252, molecular voucher No. LEP0037; **Jiangxi**: 1♂, Daqiutian, Mt. Jiulianshan, alt. 500 m, 31.VIII.2007, Zhang Dandan leg., genitalia slide No. HFX08056; 2♂5♀, Mt. Jiulianshan, Longnan, 24.58°N, 114.43°E, alt. 620 m, 26.IX.2016, Chen Kai, Duan Yongjiang leg., genitalia slide No. SYSU1546 (♂); 1♀, Mt. Jinggangshan, 2.X.2010, Chen Haidong, Xie Weicai leg., genitalia slide No. SYSU0216; **Shanxi**: 2♂, Dahe Forestry Station, Yicheng, 35.46°N, 111.93°E, alt. 1212m, 13–15.VIII.2018, Xiang Lanbin leg., genitalia slide No. SYSU1542; 2♂, Miaoping, Manghe, Yangcheng, 35.25°N, 112.46°E, alt. 557 m, 19–20.VIII.2018, Xiang Lanbin leg., genitalia slide No. SYSU1545.

#### Remarks.

*Ecpyrrhorrhoepuralis* is mainly reported from central and southern China and Japan. Solis et al. (2010) considered it was introduced in eastern North America and spread following the invasive host *Paulowniatomentosa* (Thunb.) Steud.

#### Distribution.

China (Guangdong, Guangxi, Hebei, Henan, Hubei, Hunan, Jiangxi, Shandong, Shanxi), Japan, India, North America.

### ﻿ Ecpyrrhorrhoe rubellalis

Taxon classificationAnimaliaLepidopteraCrambidae

(Snellen, 1890) comb. nov.

D74BE70A-6B0E-53D6-B5DE-D43F58B7FB6C

Figs 18 , 34 , 49

Botys rubellalis Snellen, 1890: 577.
Ecpyrrhorrhoe aduncis Gao, Zhang & Wang, 2013: 312. Syn. nov.

#### Diagnosis.

**Figures 34–37.**
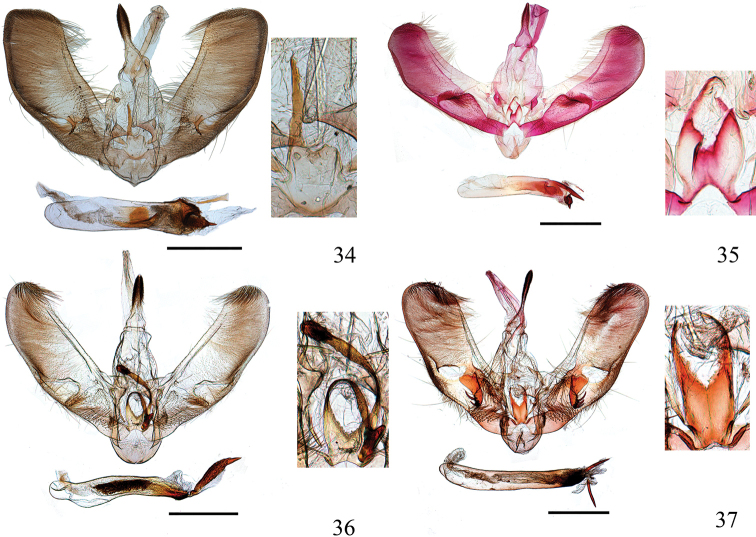
Male genitalia of *Ecpyrrhorrhoe* spp., with enlarged detail of juxta (right) **34***E.rubellalis*, Hainan (genitalia slide No. SYSU0230) **35***E.longispinalis* sp. nov., holotype, Hunan (genitalia slide No. CXH12194) **36***E.celatalis*, Hainan (genitalia slide No. SYSU0242) **37***E.biaculeiformis*, Fujian (genitalia slide No. SYSU0261). Scale bars: 1.0 mm.

Forewing length: 11.0–14.0 mm. In appearance, *Ecpyrrhorrhoerubellalis* resembles *E.minnehaha*, but can still be recognized by its larger forewing length, yellowish brown ground color of wings with yellow fringe, and more oblique antemedial line of forewing (Fig. 18); in the male genitalia (Fig. 34) by the broader valva with truncate tip, and the nearly spine-shaped, thin and short sella, by the phallus apically with a densely dentated, triangular projection and a thick spine bearing a broad and long, spinulose base on the anellus; in the female genitalia (Fig. 49) by the antrum without spinules and large second (posterior) signum.

#### Material examined.

**Type material.***Botysrubellalis*: ***Lectotype*** (newly designated in this study) ♂, Sikkim, ?000 feet, 1886, O. Möller [leg.], NHMUK slide No. 010315144 (NHMUK). *Ecpyrrhorrhoeaduncis*: ***Holotype*** ♂, **China: Taiwan**: Sikanshui, Taipei, 25.01° N, 121.27°E, alt. 550–600 m, 4.VIII.2006, Li Houhun leg., genitalia slide No. GQ11127 (NKU).

#### Other material examined.

**China: Guangxi**: 1♀, Hekou Station, Mt. Dayaoshan, 24.14°N, 110.09°E, alt. 823 m, 18.VII.2015, Zhao Shengnan, Qi Mujie leg. (NKU); 2♂, Lianhuashan, Mt. Dayaoshan, 24.15°N, 110.11°E, alt. 1000 m, 22.VII.2015, Zhao Shengnan, Qi Mujie leg., genitalia slide No. ZDD12046, molecular voucher No. LEP0110 (NKU); 1♂, Bainan, Napo, 23.03°N, 105.48°E, alt. 300 m, 23.VII.2013, Chen Xiaohua leg., genitalia slide No. SYSU1561; **Hainan**: 2♂1♀, Mt. Yinggeling, 19.05°N, 109.50°E, alt. 954 m, 4.IX.2013, Chen Xiaohua leg., genitalia slide No. SYSU0008 (♂), SYSU0230 (♂), SYSU0243 (♀, molecular voucher No. LEP0023); 1♂, Yingzui, Mt. Yinggeling, 23.V.2010, Du Xicui, Liao Li leg., genitalia slide No. SYSU1562; 1♂2♀, Shuiman Village, Mt. Wuzhishan, 18.88°N, 109.67°E, alt. 667 m, 6.IX.2013, Li Jinwei, Chen Xiaohua, Xie Weicai leg.; 1♂, Mt. Wuzhishan, 18.53°N, 109.39°E, alt. 742 m, 22.V.2015, Cong Peixin, Guan Wei, Hu Sha. leg. (NKU); 1♂1♀, Mt. Wuzhishan, alt. 795 m, 19, 20.V.2014, Xu Lijun, Xu Dan leg.; 1♂, Mt. Diaoluoshan, alt. 500 m, 24.V.2014, Xu Lijun, Xu Dan leg.; 1♀, Mt. Diaoluoshan, 18.43°N, 109.52°E, alt. 922 m, 26.V.2015, Cong Peixin, Guan Wei, Hu Sha. leg. (NKU); 1♀, Nankai Village, Baisha, 19.07°N, 109.42°E, alt. 294 m, 19.V.2013, Li Jinwei leg.; 1♀, Mt. Limushan, 19.18°N, 109.73°E, alt. 755 m, 2.XI.2013, Chen Kai, Chen Xiaohua leg.

#### Distribution.

China (Guangxi, Hainan, Taiwan), India.

#### Remarks.

Snellen (1890) described *E.rubellalis* from two specimens collected by Möller in Sikkim. One of them is here designated as the lectotype.

### ﻿ Ecpyrrhorrhoe longispinalis

Taxon classificationAnimaliaLepidopteraCrambidae

Zhang & Xiang sp. nov.

C14479A9-D981-524C-8084-F65C7D6F5D09

http://zoobank.org/746AB7EC-3DED-43A3-8EDE-DBB26AB448A1

Figs 19 , 35 , 50

#### Diagnosis.

*Ecpyrrhorrhoelongispinalis* can be distinguished from *E.digitaliformis* and *E.puralis* in the male genitalia (Fig. 35) by the valva gradually broadening to the sub-apex, a hook-shaped sella, a small, sclerotized, ball-shaped sclerite bearing two small spines on opposite sides on the anellus; in the female genitalia (Fig. 50) by the antrum without sclerotized processes or triangular, wrinkled sclerites.

#### Description

(Fig. 19). **Head.** Frons pale yellow, with white lateral bands. Vertex pale yellow. Labial palpus dark yellow, contrastingly white at base ventrally. Maxillary palpus dark yellow, pale terminally. **Thorax.** Dorsal side dark yellow or yellowish brown, ventral side white. Legs white to pale yellow. **Wings.** Forewing length: 9.0–13.0 mm. Forewing bright yellow, termen moderately arched; antemedial line fulvous, outwardly curved from 1/4 of costa to 1/3 of posterior margin; orbicular stigma dot-shaped, small, sometimes indistinct; reniform stigma comma-shaped, concave; postmedial line from anterior 3/4 distinctively curved to middle of CuA_1_, then bending to 1/3 of CuA_2_, and finally undulated to 2/3 of posterior margin; terminal line and fringe bright yellow. Hindwing yellow, costal area white, postmedial line fulvous, slightly dentate curved, outward from 2/3 of M_1_ to 1/2 of CuA_1_, arc-shaped, then bending inward along CuA_1_, reaching discocellular, then undulated to 2/3 of posterior margin; terminal line and fringe as in forewing. **Abdomen.** Pale yellow dorsally, black on distal part, white ventrally.

#### Male genitalia

**(Fig. 35).** Uncus relatively thick, with basal half nearly triangular and naked. Valva curved and slowly broadening to rounded apex, with maximal width at sub-apex; sella hook-shaped with basal half densely setose; sacculus with distal 3/5 inflated into a triangular, rounded protrusion. Juxta with basal margin concave, distal half bifid into stout and pointed arms; anellus bearing a small and sclerotized ball, with two small spines on opposite sides (attached to distal end of phallus in Fig. 35). Saccus rounded triangular. Phallus long and slightly curved, cornuti presented as a long sclerite and a long and strong spine on apical end.

#### Female genitalia

**(Fig. 50).** Anterior apophysis ~ 2× length of posterior apophysis. Lamella postvaginalis presented as a nearly trapezoidal sclerite. Antrum cup-shaped, strongly sclerotized, decorated with lots of small spines, those spines forming a circle, with a thumb-shaped, sclerotized process on the side of circle; colliculum narrow and moderately long; ductus seminalis connecting to anterior end of colliculum and with a short sclerite located opposite to colliculum; ductus bursae slender, length ~ 2× as long as diameter of corpus bursae, basal 1/3 bearing a slim sclerite. Corpus bursae globular; rhomboid signum with maximal length almost 1/3 as long as diameter of corpus bursae; second (posterior) signum nearly V-shaped bearing sparse and long spines.

#### Material examined.

***Holotype*** ♂, **China: Hunan**: Zhupo Village, Huitong, 23.VIII.2012, Li Jinwei, Chen Xiaohua leg., genitalia slide No. CXH12194 (SYSBM). ***Paratypes*: China: Hubei**: 1♀, Taohuachong, Mt. Dabieshan, 30.59°N, 116.19°E, alt. 661 m, 24.VI.2014, Chen Xiaohua, Pan Chang leg., genitalia slide No. SYSU1541; **Hunan**: 1♂1♀, same data as holotype, genitalia slide No. SYSU0301 (♂, molecular voucher No. LEP0401), CXH12200 (♀, molecular voucher No. LEP0058).

#### Distribution.

China (Hubei, Hunan).

#### Etymology.

The specific name is derived from the combination of Latin *long*- and *spinalis* (= with spine), referring to the vesica with a long and thick spine.

### ﻿ Ecpyrrhorrhoe celatalis

Taxon classificationAnimaliaLepidopteraCrambidae

(Walker, 1859)

39826838-B973-5531-A062-E4B140EEDBED

Figs 20 , 36 , 51

Botys celatalis Walker, 1859: 657.
Botys rhoecusalis Walker, 1859: 1000.
Pyrausta retostalis E. Hering, 1901: 54–56.
Ecpyrrhorrhoe ruidispinalis Zhang, Li & Wang, 2004: 322. Syn. nov.

#### Diagnosis.

Forewing length: 10.0–13.0 mm. *Ecpyrrhorrhoecelatalis* can be differentiated from *E.digitaliformis* and *E.brevis* in the male genitalia (Fig. 36) by the straight costa of the valva, the extremely long arms of the juxta with an apical, large, and sclerotized tooth, and the anellus with a long, thick and large spine, and decorated with many tiny spines on its basal 3/4 (attached to distal end of phallus in Fig. 36), in the female genitalia (Fig. 51) by the posterior part of the antrum looking like a pair of triangular sclerites.

**Figures 38–43.**
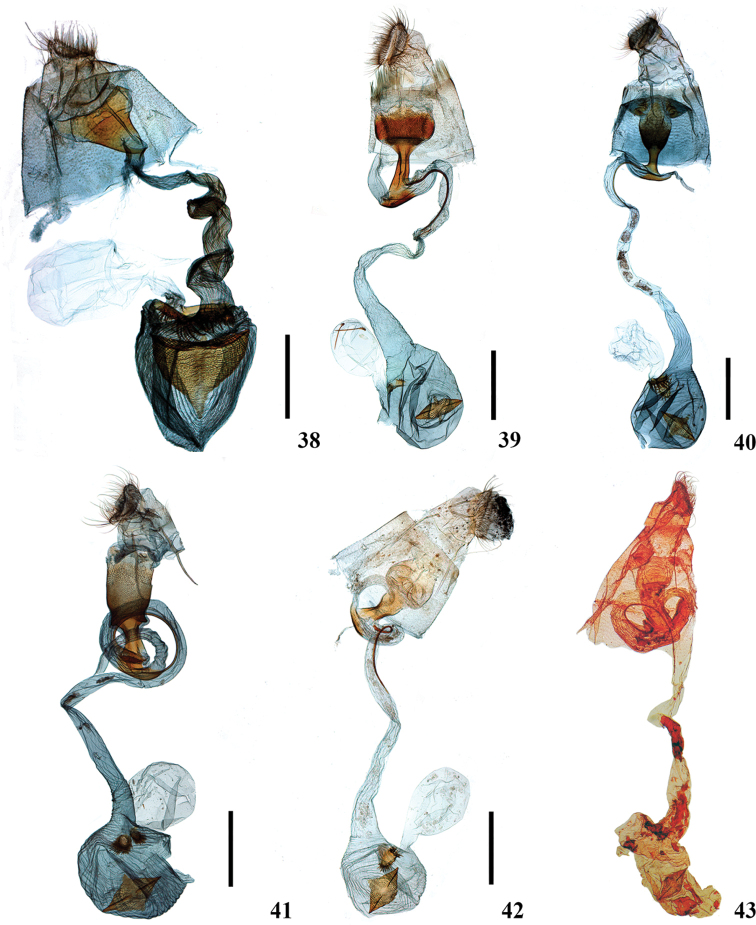
Female genitalia of *Ecpyrrhorrhoe* spp. **38***E.damastesalis*, Hainan (genitalia slide No. SYSU0247) **39***E.minnehaha*, Shaanxi (genitalia slide No. SYSU0272) **40***E.obliquata*, Guangdong (genitalia slide No. SYSU0269) **41***E.rufipicta*, Hainan (genitalia slide No. SYSU0341) **42***E.rubiginalis*, Jiangxi (genitalia slide No. SYSU0215) **43***E.machoeralis*, type, Ceylon (genitalia slide No. 8680 (NHMUK)). Scale bars: 1.0 mm.

#### Material examined.

*Botyscelatalis*: ***Type***: 1♀, Ceylon, Pyralidae Brit. Mus. Slide No. 8686 (NHMUK). *Ecpyrrhorrhoeruidispinalis*: ***Holotype*** ♂, **China: Guangxi**: Shangsi, 22.09°N, 107.58°E, alt. 770 m, 3.IV.2002, Hao Shulian, Xue Huaijun leg., genitalia slide No. ZDD02357 (NKU).

#### Other material examined.

**India**: 1♂, Khasi Hills, 5.3[III].1972, S. N. A. Jacobs, Pyralidae Brit. Mus. Slide No. 010891075 (NHMUK). **China: Chongqing**: 1♀, Mt. Jinfoshan, alt. 1100 m, 4.VIII.2012, Yang Xiaofei, Liu Tengteng leg., genitalia slide No. SYSU1530 (NKU); **Fujian**: 1♂, Letu Village, Nanjing, 24.90°N, 117.22°E, alt. 270 m, 10.VII.2014, Zhang Dandan leg., genitalia slide No. SYSU0232; 1♀, Mt. Tianzhushan, 24.VII.2014, Yang Xiaofei leg., genitalia slide No. ZDD12028 (NKU); **Guangdong**: 3♂2♀, Mt. Danxiashan, Shaoguan, 25.04°N, 113.64°E, alt. 96 m, 6–7.VI.2012, Li Jinwei leg., genitalia slide No. SYSU0249(♂), SYSU0250(♀), CXH12173(♂), ZDD10072(♀); 5♀, Guangzhou, 1948, genitalia slide No. ZDD03025; 1♂, Dawuling, Xinyi, alt. 900 m, 9.VIII.2003, Zhang Dandan, Li Zhiqiang leg., genitalia slide No. ZDD03018; 1♀, Neilingding Island, Shenzhen, 5.VII.1998, Wen Ruizhen leg., genitalia slide No. ZDD03024; 1♂, Mt. Nankunshan, Huizhou, 16.VII.2003, Zhang Dandan, Li Zhiqiang leg., genitalia slide No. ZDD03022; 1♀, Bijialing, Mt. Potoushan, Zhanjiang, 4.VI.2016, Li Zhiqiang leg., genitalia slide No. SYSY0264, molecular voucher No. LEP0400; 1♂, Heishiding, Fengkai, 5.IX.2011, Yang Lijun, Cheng Muchun, Liao Junlei leg., genitalia slide No. SYSU0231(♂); 1♀, Yanshuitian, Fengkai, 3.VI.2011, Chen Haidong, Tong Bo leg., genitalia slide No. SYSU0220; 1♂, Lianping, 12.VIII.2009, Zeng Yanyi leg., genitalia slide No. SYSU1534; **Guangxi**: 6♂, Huaping National Natural Reserve, Guilin, 25.63°N, 109.91°E, alt. 520 m, 11–12.IX.2020, Jin Mengjie, Xiang Lanbin leg.; 3♂1♀, Technology Building, Huaping Natural Reserve, Guilin, 25.63°N, 109.91°E, alt. 760 m, 10–12.IX.2020, Jin Mengjie, Xiang Lanbin leg.; 5♂3♀, the lookout, Huaping Natural Reserve, Guilin, 25.61°N, 109.90°E, alt. 950 m, 10.IX.2020, Jin Mengjie, Xiang Lanbin leg.; 3♂1♀, Nonggang, Longzhou, 22.47°N, 106.96°E, alt. 271 m, 20–21.VIII.2011, 19.VI.2012, Li Jinwei, Cheng Muchun leg., genitalia slide No. SYSU0052(♂), SYSU0223(♀), CXH12191(♂); 1♀, Jinxiazhai, Mulun Natural Reserve, 22.47°N, 106.96°E, alt. 288 m, 19.VII.2015, Xu Dan Leg., genitalia slide No. SUSU0307; 1♀, Yangmeiao, Mt. Jiuwandashan, 25.19°N, 108.65°E, alt. 1183 m. 22.VII.2015, Chen Kai leg., genitalia slide No. SYSU0306, molecular voucher No. LEP0403; 1♀, Mt. Jinzhongshan, 24.67°N, 104.88°E, alt. 957 m, 18.VII.2013, Chen Xiaohua leg., genitalia slide No. SYSU1526; 1♂, Gaozhai, Mt. Maoershan, Guilin, alt. 1100 m, 27.VII.2015, Liu Kaili, Zhao Jingxia leg., genitalia slide No. SYSU1531; **Hainan**: 2♂1♀, Yinggeling Natural Reserve, 19.05°N, 109.50°E, alt. 954 m, 4.IX.2013, Xie Weicai, Chen Xiaohua leg., genitalia slide No. CXH12188(♂), SYSU0242(♂, molecular voucher No. LEP0017), SYSU1536(♀); 1♂, Bangxi Natural Reserve, 19.37°N, 109.10°E, alt. 97 m, 2.IX.2013, Xie Weicai leg., genitalia slide No. SYSU0300; 1♀, Mt. Diaoluoshan, 18.67°N, 109.93°E, alt. 94 m, 16.V.2013, Li Jinwei Leg., genitalia slide No. SUSU0305; **Hunan**: 33♂9♀, Hydro-electric power station, Yueyan Forestry farm, Dupangling National Reserve, Yongzhou, 25.48°N, 111.36°E, alt. 430 m, 29–30.VIII.2020, Jin Mengjie, Xiang Lanbin leg., genitalia slide No. SYSU1566 (♂), SYSU1567 (♂), SYSU1569 (♂), SYSU1570 (♂), SYSU1571 (♀), SYSU1580 (♀), SYSU1581 (♂), SYSU1582 (♂), SYSU1583 (♀), SYSU1584 (♂), SYSU1585 (♀); 1♂1♀, Northeast of Yueyan Forestry farm, Dupangling National Reserve, Yongzhou, 25.49°N, 111.39°E, alt. 350 m, 28–31.VIII.2020, Jin Mengjie, Xiang Lanbin leg.; 1♀, Mt. Shunhuangshan, Xinning, Shaoyang, 26.40°N, 111.00°E, alt. 810 m, 4–6.IX.2020, Jin Mengjie, Xiang Lanbin leg.; **Jiangxi**: 1♀, Xiaoxidong, Mt. Jinggangshan, 2.VIII.2011, Li Jingwei leg., genitalia slide No. SYSU0273; 1♂, Daqiutian, Mt. Jiulianshan, Longnan, 31.VIII.2007, alt. 500 m, Jia Fenglong leg., genitalia slide No. HFX08084; **Tibet**: 1♂1♀, Medog, 29.20°N, 95.20°E, alt. 1103 m, 8.VII.2013, Li Jinwei leg., genitalia slide No. SYSU0219 (♀), SYSU1535(♂); **Yunnan**: 2♂7♀, Baihualing Natural Reserve, Baoshan, alt. 1520 m, 11, 13.VIII.2007, Zhang Dandan leg., genitalia slide No. CXH 12178(♀), SYSU0007(♂), SYSU0037(♀), SYSU0222(♂); 1♂, Baihualing Natural Reserve, Mt. Gaoligongshan, Baoshan, 24.30°N, 98.80°E, alt. 1535 m, 20. IV.2015, Chen Kai, Duan Yongjiang leg., genitalia slide No. SYSU0211; 1♀, Hanlongzhai, Baihualing, Baoshan, 25.31°, 98.80°E, alt. 1616 m, 11.V.2021, Jin Mengjie, Guo Muyu, Fu Haiyun leg., genitalia slide No. SYSU1555.

**Figures 44–49.**
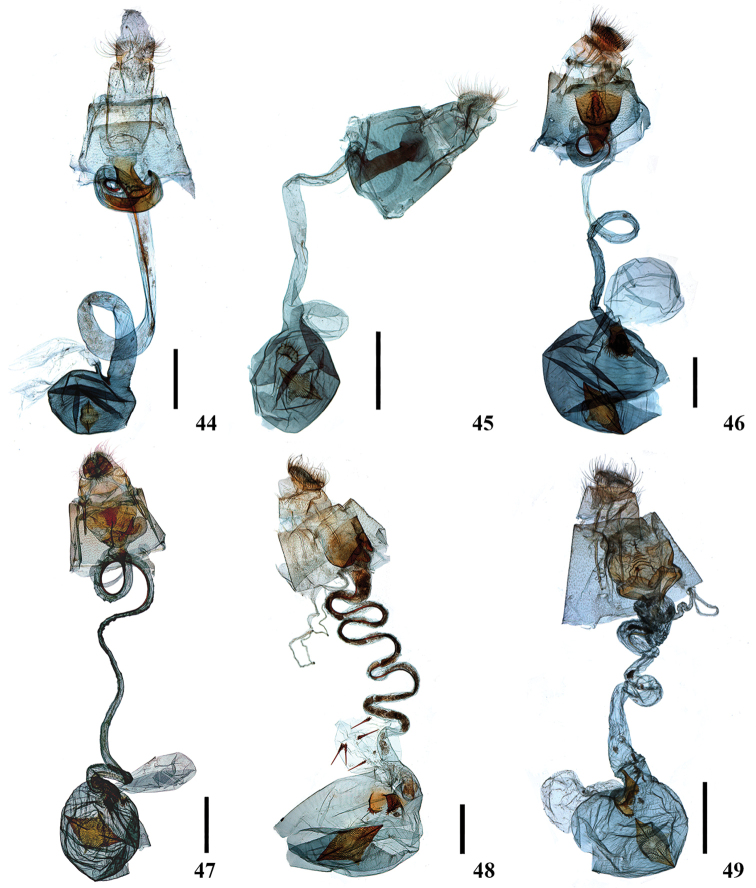
Female genitalia of *Ecpyrrhorrhoe* spp. **44***E.rosisquama* sp. nov., paratype, Yunnan (genitalia slide No. SYSU0262) **45***E.exigistria* sp. nov., paratype, Jiangxi (genitalia slide No. SYSU0276) **46***E.digitaliformis* (genitalia slide No. SYSU0274) **47***E.brevis* sp. nov., paratype, Guangdong (genitalia slide No. SYSU1533) **48***E.puralis*, Jiangxi (genitalia slide No. SYSU0216) **49***E.rubellalis*, Hainan (genitalia slide No. SYSU0243). Scale bars: 1.0 mm.

#### Distribution.

China (Chongqing, Fujian, Guangdong, Guangxi, Hainan, Hunan, Jiangxi, Tibet, Yunnan), India, Sri Lanka.

### ﻿ Ecpyrrhorrhoe biaculeiformis

Taxon classificationAnimaliaLepidopteraCrambidae

Zhang, Li & Wang, 2004

0AAD95CA-EF91-5076-9009-415087D2FDCA

http://zoobank.org/669B6A2F-ED99-4B4B-A145-5DED918C2DFE

Figs 21 , 37 , 52

Ecpyrrhorrhoe biaculeiformis Zhang, Li & Wang, 2004: 317.

#### Diagnosis.

Forewing length: 12.0–16.0 mm. *Ecpyrrhorrhoebiaculeiformis* is similar to *E.puralis* in habitus, but can be distinguished by the much larger size and much paler coloration (Fig. 21), in the male genitalia (Fig. 37) by the width of valva relatively even or slowly widening distally, a sella with the basal part bearing 4 spines on the apex and with a distal process, the narrow and short juxta with distal half bifid, the anellus with two separate spines (attached to distal end of phallus in Fig. 37); in the female genitalia (Fig. 52) by the antrum bearing a pair of nearly triangular and wrinkled sclerites at posterior margin, and the short sclerite of ductus bursae ~ 2/5 of its length.

#### Material examined.

***Holotype*** ♂, **China: Guizhou**: Mt. Fanjingshan, 27.55°N, 108.41°E, alt. 1300 m, 2.VIII.2001, Li Houhun, Wang Xinpu Leg., genitalia slide No. ZDD02119 (NKU).

#### Other material examined.

**China: Anhui**: 2♂1♀, Tangkou, Mt. Huangshan, 30.05°N, 118.11°E, alt. 580 m, 19–20.IX.2012, Yang Lijun leg., genitalia slide No.SYSU1515(♂), CXH12205 (♀, molecular voucher No. LEP0397); **Fujian**: 1♂, Tongmu, Mt. Wuyishan, 27.75°N, 117.68°E, alt. 759 m, 19.V.2012, Li Jinwei leg., genitalia slide No. SYSU0261; **Guangdong**: 2♂1♀, Heishiding, Fengkai, 7.V, 9.X.2010, 1.V, 5.IX, 5.X.2011, Zhang Dandan, Tong Bo, Chen Haidong, Jin Zhenyu, Li Yun leg., genitalia slide No. CXH12171 (♂), CXH12184 (♂), CXH12204 (♀); 1♂, Lianping, 12.VIII.2009, Zeng Yanyi leg., genitalia slide No. CXH12202; 1♂1♀, Mt. Nankunshan, Huizhou, 16.VII.2003, Zhang Dandan, Li Zhiqiang leg., genitalia slide No. ZDD03019 (♀), ZDD03020 (♂); **Guangxi**: 5♂2♀, Gaozhai Village, Xing’an, 28.VIII.2011, Zhang Dandan, Li Jinwei leg., genitalia slide No. SYSU0204(♂), CXH12168(♂); 1♂, Anjiangping Natural Reserve, 25.56°N, 109.93°E, alt. 1751 m, 10.VII.2013, Chen Xiaohua leg.; 1♀, Yinshan Natural Reserve, Jinxiu, 24.15° N, 110.21°E, alt. 1464 m, 8.VII.2013, Chen Xiaohua leg.; **Guizhou**: 1♀, Maolan Reserve, 1.IX.2011, Li Jinwei leg., genitalia slide No. CXH12199; 1♂, Taojiang, Leishan, 27.VIII.2012, Li Jinwei, Chen Xiaohua leg.; 1♂, Weng’ang, Maolan Reserve, Libo, 25.25°N, 107.90°E, alt. 814 m, 25.VII.2015, Chen Kai leg., genitalia slide No. SYSU0255; **Hubei**: 2♀, Maoping Village, Wufeng, 30.08°N, 110.40°E, alt. 1175 m, 11.IX.2012, Li Jinwei leg.; 1♂, Qingtaiguan, Luotian, 31.11°N, 115.41°E, alt. 524 m, 2.VII.2014, Liu Zhenhua, Pan Chang leg.; 1♂, Tiantangzhai, Luotian, 31.06°N, 115.44°E, alt. 570 m, 17.IX.2012, Yang Lijun leg.; 1♂, Wujiashan, Yingshan, 31.05°N, 115.47°E, alt. 880 m, 29.VI.2014, Chen Xiaohua, Pan Chang leg., genitalia slide No. SYSU0244, molecular voucher No. LEP0024; **Hunan**: 5♂4♀, Zhangjiajie Forest Park, 29.18°N, 110.26°E, alt. 625 m, 13.IV.2012, Li Jinwei, Yang Lijun leg., genitalia slide No. SYSU0012(♂); 1♂1♀, Mt. Tianzishan, Zhangjiajie, 29.23°N, 110.29°E, alt. 1096 m, 14.IX.2012, Li Jinwei, Yang Lijun leg., genitalia slide No. CXH12158(♂); 3♀, Zhupo Village, Huitong, 23.VIII.2012, Li Jinwei, Chen Xiaohua leg., genitalia slide No. CXH12201, CXH12219, SYSY0302; 2♂, Jinyinpu, Bamianshan Natural Reserve, Guidong, 25.97°N, 113.71°E, alt. 973 m, 16.VI.2015, Chen Kai leg., genitalia slide No. SYSU0240, molecular voucher No. LEP0015; 5♂1♀, Mt. Huilongshan, Zixing, 26.08°N, 113.39°E, alt. 886 m, 17.IX.2017, Chen Kai leg., genitalia slide No. SYSU1521(♂); 1♂, Shennonggu Forest Park, Yanling, 26.52°N, 114.01°E, alt. 379 m, 17.VI.2017, Chen Kai leg., genitalia slide No. SYSU1520; **Jiangxi**: 4♂2♀, Qianmo Village, Nanfengmian Nature Reserve, Suichuan, 26.28°N, 114.06°E, alt. 816 m, 19.VI.2015, Chen Kai leg.; 3♂, Qianmo Village, Nanfengmian Nature Reserve, Suichuan, 26.29°N, 114.06°E, alt. 820 m, 19.IX.2017, Chen Kai leg.; 2♂1♀, Mt. Guanggushan, Wuzhifeng, Shangyou, 25.92°N, 114.05°E, alt. 846 m, 22.VI.2015, Chen Kai leg., genitalia slide No. SYSU0203(♂); 4♂1♀, Mt. Guanggushan, Shangyou, 25.92°N, 114.05°E, alt. 183 m, 20.IX.2016, Chen Kai, Duan Yongjiang leg.; 1♂, Zaodu Village, Nanshan, 29.01°N, 115.16°E, alt. 315 m, 19.VII.2014, Chen Kai leg.; 1♂, Guanyinyan, Jing’an, 29.03°N, 115.25°E, alt. 195 m, 20.VII.2014, Chen Kai leg.; 9♂, Daqishan Forestry Station, Jing’an, 28.67°N, 115.07°E, alt. 350 m, 16.VII.2014, Chen Kai leg., genitalia slide No. SYSU0256; 6♂, Xiaoxidong, Mt. Jinggangshan, 1–2.VII.2011, Xie Weicai leg., genitalia slide No. CXH12203, CXH12207, CXH12210; 4♂4♀, Zhufeng, Mt. Jinggangshan, 28.IV, 30.VI, 3.VIII, 1.IX.2011, Li Jinwei, Mei Yan, Liu Ping, Cheng Muchun leg., genitalia slide No. CXH12208(♀), CXH12215(♀); 2♀, Luofu, Mt. Jinggangshan, 27.IV, 3, 30.VIII.2011, Li Jinwei, Cheng Muchun leg., genitalia slide No. CXH12217; 1♂, Luofu, Mt. Jinggangshan, 18.IX.2010, Zhang Dandan, Zhao Shuang, Tong Bo leg., genitalia slide No. CXH12206; 1♂3♀, Mt. Jiulianshan, Longnan, 24.58°N, 114.43°E, alt. 620 m, 26.IX.2016, 24.IX.2017, Chen Kai, Duan Yongjiang leg., genitalia slide No. SYSU1525(♂), SYSU1548(♀); 2♂, Guanshan National Natural Reserve, Yifeng, 28.55°N, 114.58°E, alt. 394 m, 14.VI.2016, Chen Kai, Duan Yongjiang leg., genitalia slide No. SYSU1519(♂); 1♀, Shixi Village, Fengxin, 28.44°N, 114.54°E, alt. 506 m, 22.IX.2012, Li Jinwei leg; **Shaanxi**: 1♂, Huoditang Forestry Station, Ningshan, 33.43°N, 108.45°E, alt. 1497 m, 29–31.VII.2018, Liu Qingming, Xiang Lanbin leg., genitalia slide No. SYSU1516; 2♀, Yueba, Foping, 33.55°N, 107.82°E, alt. 1052 m, 1–3.VIII.2018, Liu Qingming, Xiang Lanbin leg., genitalia slide No. SYSU1517; **Sichuan**: 8♂4♀, Xixi Village, Huagaoxi, alt. 1181 m, 10–13.IX.2014, Xu Dan, Wei Xuli leg., genitalia slide No. SYSU0303(♂), SYSU1523(♂); 1♂, Reserve Station of Huagaoxi, alt. 621 m, 5.IX.2014, Xu Dan, Wei Xuli leg., genitalia slide No. SYSU1524(♂); 2♂, Guandou Village, Huagaoxi, alt. 763 m, 30.VIII, 2.IX.2014, Xu Lijun, Xu Dan, Wei Xuli leg.; 1♂, Dahonghai, Mt. Simianshan, alt. 1120 m, 17.VII.2010, Du Xicui, Song Lifang leg.; **Zhejiang**: 1♂, Mt. Tianmushan, Lin’an, 30.31°N, 119.44°E, alt. 295 m, 11.V.2012, Li Jinwei leg., genitalia slide No. SYSU0097.

**Figures 50–52.**
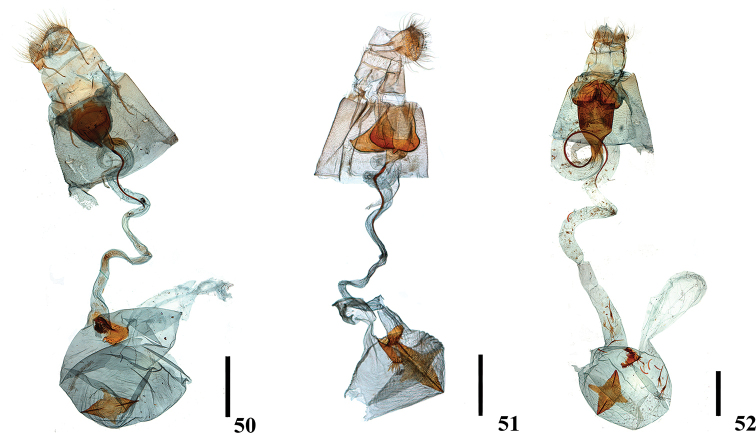
Female genitalia of *Ecpyrrhorrhoe* spp. **50***E.longispinalis* sp. nov., paratype, Hunan (genitalia slide No. CXH12200) **51***E.celatalis*, Fujian (genitalia slide No. ZDD12028) **52***E.biaculeiformis* (genitalia slide No. CXH12217). Scale bars: 1.0 mm.

#### Distribution.

China (Anhui, Fujian, Guangdong, Guangxi, Guizhou, Hubei, Hunan, Jiangxi, Shaanxi, Sichuan, Zhejiang).

## ﻿Discussion

Based on the results of the phylogenetic analysis and the presence of specialized sclerotized structures on the anellus, *Paliga* is here treated as a new synonym of *Ecpyrrhorrhoe*. Based on the examination of type material, seven species of *Paliga*, *P.auratalis* (Warren, 1895), *P.damastesalis* (Walker, 1859), *P.machoeralis* (Walker, 1859), *P.minnehaha* (Pryer, 1877), *P.rubicundalis* Warren, 1896, *P.rufipicta* (Butler, 1880) and *P.schenklingi* Strand, 1918 are confirmed to belong in *Ecpyrrhorrhoe*. In the case of *P.anpingialis* Strand, 1918, the female genitalia of the holotype (♀, Anping, Formosa, IV.1912, H. Sauter Coll., Gen. präp. Gaedike NR: 9668 (SDEI)) does not have the diagnostic characters of *Ecpyrrhorrhoe* (absence of lamella antevaginalis, longitudinal stripe on ductus bursae, and second (posterior) signum) and is not congeneric with *Ecpyrrhorrhoe*, but its correct placement is unclear due to the lack of male material. The abdomens of the types of *P.leucanalis* Swinhoe, 1890 and *P.suavalis* (Walker, 1866) are lost. The genitalia slide of the type of *P.fuscicostalis* Swinhoe, 1894 is incorrect and may have been confused with that of Pyralidae Brit. Mus. Slide No. 8683, which is labelled with “incorrect abdomen? See 8683 for correct abdomen”. The types of *P.quadrigalis* (Hering, 1901) and *P.ignealis* (Hampson, 1899) were not examined. Therefore, these six species are transferred to *Ecpyrrhorrhoe* temporarily, with their generic placement unconfirmed. Further study is needed to confirm their generic placement.

Also, based on our phylogenetic results and study of genitalic characters, another three species, *Ananiafimbriata* (Moore, 1886), *Ananiaobliquata* (Moore, 1888) and *Pyraustarubellalis* (Snellen, 1890) are placed in *Ecpyrrhorrhoe*.

The genus *Yezobotys* Munroe & Mutuura, 1969 differs significantly in structure from *Ecpyrrhorrhoe*, and is more closely related to *Anamalaia* Munroe & Mutuura, 1969, based on examination the paratype material of *Yezobotysainualis* Munroe & Mutuura, 1969 (Pyralidae Brit. Mus. Slide No. 19693 (NHMUK)). The generic characters of *Yezobotys*, the short and triangular uncus, the sacculus with finger-shaped process in male genitalia, and the strongly sclerotized lamella antevaginalis and postvaginalis in female genitalia, are extremely similar to those of *Anamalaia* Munroe & Mutuura. Thus, *Yezobotys* is restored as a valid genus.

According to the tree topology (Fig. 1), the results of the phylogenetic analyses robustly support the monophyly of *Ecpyrrhorrhoe* in BI, but there is low support in ML (PP = 0.99, BS = 63) possibly caused by the missing data in the concatenated dataset. The genus *Pagyda* is the sister group of *Ecpyrrhorrhoe* (PP = 0.99, BS = 67), and *Ecpyrrhorrhoe* can be divided into three species groups (A clade, B clade and C clade), the B clade and C clade forming a sister group (PP = 1, BS = 49). The A clade (PP = 0.99, BS = 36), consisting of *E.allochroa* + *E.damastesalis*, can be distinguished from species in B clade and C clade by the following morphological characters: hindwing yellowish white without any lines or spot, instead of the brown postmedial line present on B clade and C clade; bifid arms of juxta short in male genitalia, ductus bursae without a slender, sclerotized, longitudinal sclerite in female genitalia. The B clade (PP = 0.95, BS = 29), consisting of seven species, can be differentiated by the transverse sclerite on the bottom of ductus seminalis in female genitalia. *E.machoeralis*, without molecular data and phylogenetic analysis, is assigned to B clade on the basis of morphological characters. The C clade (PP = 0.96, BS = 71), consisting of seven species, can be distinguished from species in A clade by a sclerotized and longitudinal stripe on ductus bursae in female genitalia, and distinguished from species in B clade by the absence of transverse sclerite on the base of ductus seminalis.

In this study, bootstrap values of the majority of the basal nodes are relatively low. Future research might utilize a broader sampling per species, fresher material more suitable for DNA studies, and additional genetic data to shed further light onto the phylogenetic relationships of this species complex.

## Supplementary Material

XML Treatment for Ecpyrrhorrhoe

XML Treatment for Ecpyrrhorrhoe allochroa

XML Treatment for Ecpyrrhorrhoe damastesalis

XML Treatment for Ecpyrrhorrhoe minnehaha

XML Treatment for Ecpyrrhorrhoe obliquata

XML Treatment for Ecpyrrhorrhoe rufipicta

XML Treatment for Ecpyrrhorrhoe fimbriata

XML Treatment for Ecpyrrhorrhoe rubiginalis

XML Treatment for Ecpyrrhorrhoe machoeralis

XML Treatment for Ecpyrrhorrhoe rosisquama

XML Treatment for Ecpyrrhorrhoe exigistria

XML Treatment for Ecpyrrhorrhoe digitaliformis

XML Treatment for Ecpyrrhorrhoe brevis

XML Treatment for Ecpyrrhorrhoe puralis

XML Treatment for Ecpyrrhorrhoe rubellalis

XML Treatment for Ecpyrrhorrhoe longispinalis

XML Treatment for Ecpyrrhorrhoe celatalis

XML Treatment for Ecpyrrhorrhoe biaculeiformis

## Supplementary materials

Supplementary material 1 Table S1 Data type Table Explanation note Pairwise distance of the COI barcoding region based on Kimura-2-parameter model. https://binary.pensoft.net/file/662145This dataset is made available under the Open Database License (http://opendatacommons.org/licenses/odbl/1.0/). The Open Database License (ODbL) is a license agreement intended to allow users to freely share, modify, and use this Dataset while maintaining this same freedom for others, provided that the original source and author(s) are credited.Lanbin Xiang, Kai Chen, Xiaohua Chen, Yongjiang Duan, Dandan Zhang
